# Rational Design, Synthesis, Molecular Docking, and Biological Evaluations of New Phenylpiperazine Derivatives of 1,2-Benzothiazine as Potential Anticancer Agents

**DOI:** 10.3390/molecules29184282

**Published:** 2024-09-10

**Authors:** Berenika M. Szczęśniak-Sięga, Natalia Zaręba, Żaneta Czyżnikowska, Tomasz Janek, Marta Kepinska

**Affiliations:** 1 Department of Medicinal Chemistry, Faculty of Pharmacy, Wroclaw Medical University, Borowska 211, 50-556 Wroclaw, Poland; 2 Department of Pharmaceutical Biochemistry, Faculty of Pharmacy, Wroclaw Medical University, Borowska 211a, 50-556 Wroclaw, Poland; natalia.zareba@umw.edu.pl (N.Z.); marta.kepinska@umw.edu.pl (M.K.); 3 Department of Basic Chemical Sciences, Faculty of Pharmacy, Wroclaw Medical University, Borowska 211a, 50-556 Wroclaw, Poland; zaneta.czyznikowska@umw.edu.pl; 4 Department of Biotechnology and Food Microbiology, Faculty of Biotechnology and Food Science, Wroclaw University of Environmental and Life Sciences, Chełmońskiego 37, 51-630 Wroclaw, Poland; tomasz.janek@upwr.edu.pl

**Keywords:** 1,2-benzothiazine, synthesis, anticancer, topoisomerase II, molecular docking, fluorescence spectroscopy

## Abstract

The aim of this study was to obtain new, safe, and effective compounds with anticancer activity since cancer is still the leading cause of mortality worldwide. The rational design of new compounds was based on the introduction of differentially substituted phenylpiperazines into the 1,2-benzothiazine scaffold as a reference for the structures of recent topoisomerase II (Topo II) inhibitors such as dexrazoxane and XK-469. The newly designed group of 1,2-benzothiazine derivatives was synthesized and tested on healthy (MCF10A) and cancer (MCF7) cell lines, alone and in combination with doxorubicin (DOX). In addition, molecular docking studies were performed both to the DNA-Topo II complex and to the minor groove of DNA. Most of the tested compounds showed cytotoxic activity comparable to doxorubicin, a well-known anticancer drug. The compound **BS230** (3-(4-chlorobenzoyl)-2-{2-[4-(3,4-dichlorophenyl)-1-piperazinyl]-2-oxoethyl}-4-hydroxy-2*H*-1,2-benzothiazine 1,1-dioxide) showed the best antitumor activity with lower cytotoxicity towards healthy cells and at the same time stronger cytotoxicity towards cancer cells than DOX. Moreover, molecular docking studies showed that **BS230** has the ability to bind to both the DNA-Topo II complex and the minor groove of DNA. Binding of the minor groove to DNA was also proven by fluorescence spectroscopy.

## 1. Introduction

Despite enormous financial resources and efforts of scientists around the world, cancer is still the leading cause of death worldwide. In 2022, there were an estimated 20 million new cancer cases and 9.7 million deaths [1]. The estimated number of people who were alive within five years following a cancer diagnosis was 53.5 million. About one in five people develops cancer in their lifetime; approximately one in nine men and one in twelve women die from the disease. What’s more, global cancer diagnoses will reach 35 million in 2050, according to the latest estimates from the World Health Organization (WHO)—an increase of 77% from the 20 million cases diagnosed in 2022 [1]. These disturbing statistics show that medicine still does not have an effective treatment for this disease. Currently used drugs, despite different mechanisms of action, have many serious side effects, which often lead to discontinuation of therapy by the patient [2]. Additionally, resistance develops to some anticancer drugs, and the therapy becomes ineffective [3]. These data show that there is still an urgent need to find new, more effective, and safer anticancer agents. One of the main drug targets used in chemotherapy to inhibit the abnormal proliferation of cancer cells is topoisomerase [4].

Topoisomerases (Topo) are the group of enzymes that control DNA topology. Topo activity increases particularly in quickly dividing cancer cells. Topo are involved in many essential cellular processes, including DNA replication, transcription, recombination, and chromosome condensation. There are two types of topoisomerases—topoisomerase I (Topo I), which catalyzes the cleavage of a single DNA strand, and topoisomerase II (Topo II), which catalyzes the cleavage of a double strand of DNA; sub-types also exist within that classification. The proper function of topoisomerases determines correct DNA topology and correct replication and transcription processes [5].

Small molecules that target topoisomerase are divided into two classes: inhibitors and poisons. Inhibitors work by blocking the catalytic function of Topo I or Topo II, preventing Topo from attaching to DNA, which leads to inhibition of DNA replication and ultimately to the stopping of cell division. The poisons fix the Topo-DNA complex, thereby preventing the rotation of the uncleaved DNA strand or blocking the reconnection of the split ends of the DNA strand. When the replication complex comes into contact with such a fixed complex, DNA is damaged, and the cell dies by apoptosis [6]. The latter type of action, especially against Topo II, has a wider application in anticancer therapy.

**Figure 1:**
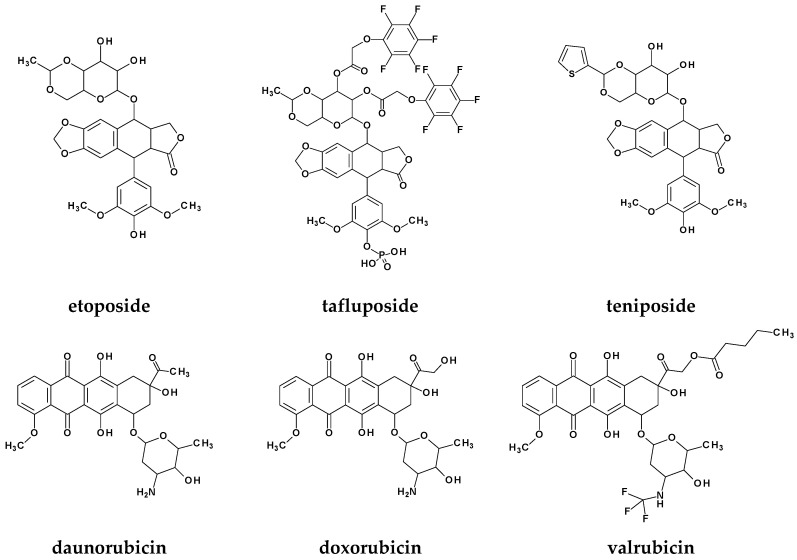

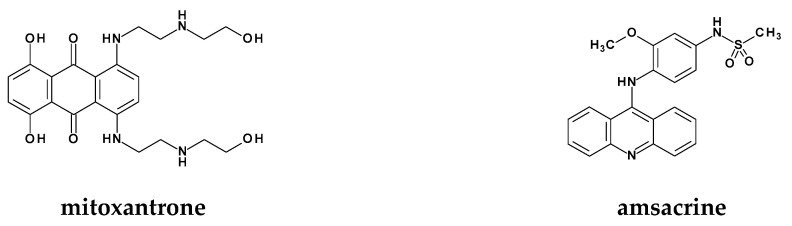
Chemical structures of selected Topo II inhibitors used as medicines.

Topo II inhibitors used as cytostatic drugs belong to several chemical groups [7]. These include lignans, which are derivatives of podophyllotoxin, such as etoposide, teniposide, and tafluposide; anthracyclines, such as daunorubicin, doxorubicin, valrubicin, epirubicin, idarubicin, and amrubicin; mitoxantrone; and amsacrine (Figure 1). Despite their effectiveness, these drugs, unfortunately, have many serious side effects, such as cardiotoxicity typical of anthracyclines or secondary leukemias after etoposide or teniposide [8].

**Figure 2:**
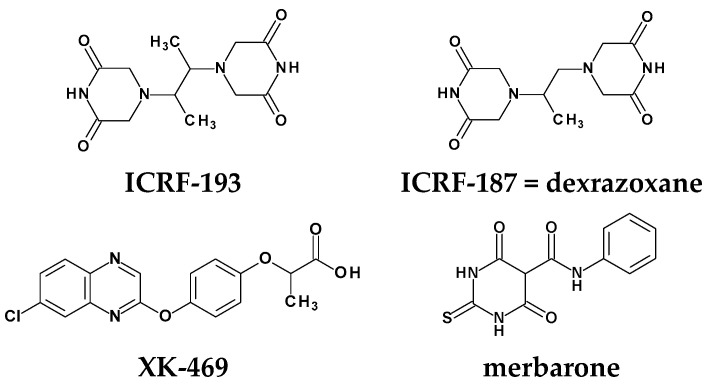
Novel Topo II inhibitors.

Novel Topo II inhibitors are also being investigated for their potential as clinically useful antineoplastic agents. The best-characterized group of compounds are bisdioxopiperazines such as ICRF-193 and ICRF-187 (dexrazoxane) (Figure 2). These compounds block the catalytic activity of DNA topoisomerase II but do not stabilize the DNA–topoisomerase II cleavable complex. Dexrazoxane, in addition to Topo inhibition, has a protective effect against cardiotoxic side effects of anthracyclines (such as doxorubicin) by preventing iron chelation by them. Moreover, during intravenous administration of anthracyclines, extravasation may occur, leading to tissue necrosis. Dexrazoxane protects tissues against symptoms of extravasation, although the mechanism of this action is unknown. Currently, dexrazoxane is a drug registered in the management and treatment of anthracycline-induced cardiotoxicity and extravasation injuries [9].

Several other compounds acting as inhibitors of Topo II, including merbarone, aclarubicin, and XK-469, have been identified (Figure 2). The most interesting compound of these three is compound XK-469 (NSC 697887), which is a synthetic quinoxaline-phenoxypropionic acid derivative that possesses unusual solid tumor selectivity and activity against multidrug-resistant cancer cells. XK-469 acts as a selective topoisomerase IIβ inhibitor. Other proposed mechanisms include XK-469-induced inhibition of cyclin B1 ubiquitination and apoptosis via binding of the peripheral benzodiazepine receptor [10,11].

Considering the urgent need for new anticancer drugs and the extremely beneficial properties of dexrazoxane and the XK-469 compound, a series of new compounds structurally related to both of them were designed as potential novel Topo II inhibitors. These new compounds were tested for their cytotoxicity to healthy and cancer cells. Additionally, the effects of the compounds in combination with DOX were tested to check whether they intensified its cytotoxic effect. Molecular dockings to both anticancer drug targets, double-stranded DNA, and DNA complexed with Topo II were also performed. Fluorescence measurements were used to prove compounds binding to DNA.

### Rational Design of New Compounds

Topoisomerase II inhibitors, as well as DNA intercalators, usually share three common essential structural features. The first one is a planar polyaromatic system that involves fused rings binding with DNA (chromophore) [12,13]. The second one is the cationic species, which increase the efficiency of DNA intercalators by interaction with the negatively charged DNA sugar–phosphate backbone, and those are basic groups: amino groups or nitrogen-containing heterocyclic compounds that can be protonated under physiological pH [14]. The third one is a groove-binding side chain, which can occupy the minor groove of DNA [15].

**Figure 3:**
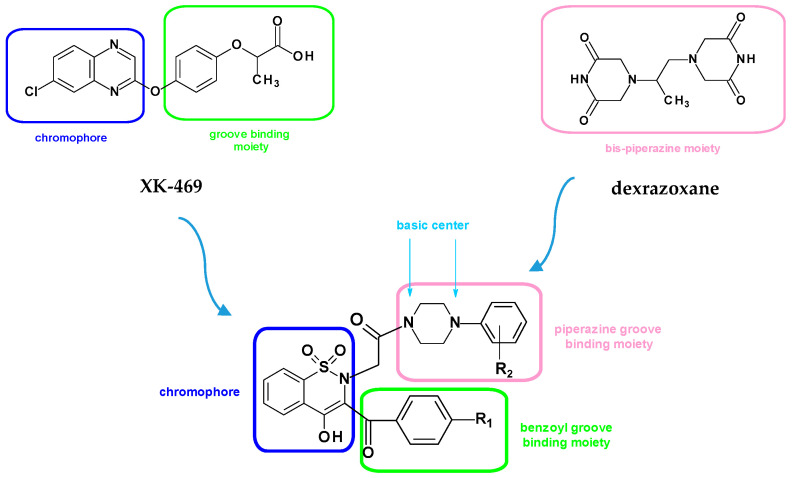
Rational design of new phenylpiperazine derivatives of 1,2-benzothiazine.

The rational design of new anticancer compounds was based on the introduction of these three essential structural elements in their structure and also on the references to the structures of dexrazoxane and XK-469 (Figure 3). The planar polyaromatic system that is a chromophore two-ring 1,2-benzothiazine scaffold was chosen because XK-469 also has a two-ring 1,4-benzodiazine chromophore. Moreover, 1,2-benzothiazine derivatives exhibit a wide spectrum of pharmacological activities, including chemopreventive and anticancer activity [16,17,18,19,20,21,22]. To this chromophore, two groove-binding side chains at positions 2 and 3 were attached to act as classical DNA intercalators. One of these is phenylpiperazine moiety, as a reference to the bis-piperazine structure of dexrazoxane. A piperazine ring has two nitrogen atoms, which are the basic centers that can be protonated under physiological pH. Furthermore, different electron-donating (CH_3_ or OCH_3_) and electron-withdrawing (Cl or F) substituents were introduced into the phenyl rings because they influence the lipophilicity of the compound and, consequently, the penetration through the nuclear membranes with the aim of a strong interaction with the DNA. The variability of substitutions also enabled to study structure–activity relationship (SAR) with the final compounds. All new compounds were divided into two series: **A** and **B**, differing in the type of substituent in position 2 of thiazine.

In general, the designed compounds were synthesized and evaluated for their *in vitro* antiproliferative activities against two human cell lines, namely, non-tumorigenic epithelial (MCF10A) and breast adenocarcinoma (MCF7) cell lines. The MCF7 breast cancer cell line was chosen for testing new compounds because in breast cancer chemotherapy, 2- and 3-drug regimens based on anthracyclines such as doxorubicin are used. The mechanism of action of anthracyclines is based on the inhibition of topoisomerase II, and the new compounds were designed as potential Topo II inhibitors. For this reason, doxorubicin was also chosen as the reference drug in these studies.

Firstly, all new compounds were tested at three different concentrations (10, 20, and 50 µM) to assess their cytotoxicity to non-tumorigenic and tumorigenic cell lines in three different time periods (24, 48, and 72 h). Secondly, all new compounds were tested with their combinations with doxorubicin (DOX) to assess if they increase the cytotoxicity of DOX, which could indicate that they reverse cell resistance to DOX. The results encouraged us to carry out further examinations to reach a deep insight into the mechanism of action of the synthesized compounds. In order to analyze the mode of binding of all compounds to DNA, fluorescence measurements were performed. Finally, molecular docking was carried out to examine the binding patterns with the prospective target, Topo II (PDBID:5GWK), and double-stranded DNA. 

## 2. Results and Discussion

### 2.1. Chemistry

**Scheme 1:**
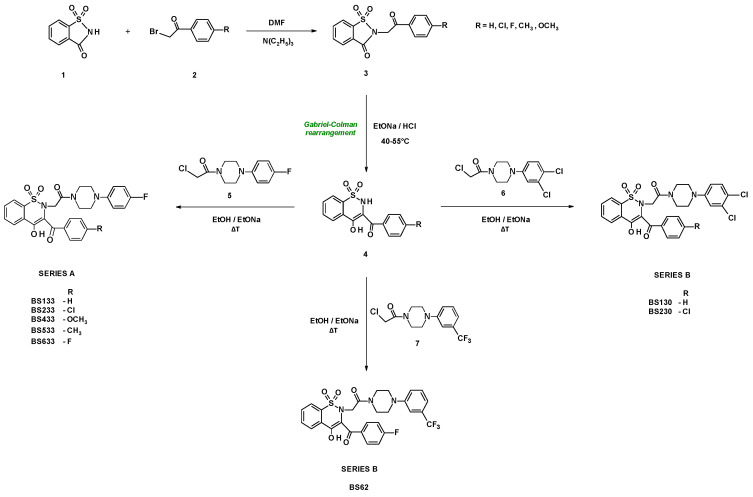
Synthesis of new phenylpiperazine derivatives of 1,2-benzothiazine.

The starting material for the synthesis of new 1,2-benzothiazine derivatives was the commercially available saccharin **1** (Scheme 1). The condensation of saccharin with various bromoacetophenones has been previously described; therefore, the steps in which compounds **3** and **4** were obtained will be described briefly [23]. In the reaction of saccharin **1** with bromoacetophenones **2** substituted in the para position with various groups (Cl, F, CH_3_, OCH_3_) or non-substituted in the environment of dimethylformamide (DMF) and triethylamine (TEA), compounds **3** were formed. These compounds were subjected to the Gabriel-Colman rearrangement reaction, as a result of which from the five-membered 1,2-thiazole ring, a six-membered 1,2-thiazine ring was formed. In this reaction, after adding a stoichiometric amount of sodium ethoxide, the 1,2-benzothiazole ring opens with the cleavage of the N-C=O bond, which then cyclizes in the presence of excess sodium ethoxide to 1,2-benzothiazine **4** (Scheme 1). The various 1,2-benzothiazine derivatives **4** thus obtained were alkylated with 1-(2-chloro-1-oxoethyl)-4-(p-fluorophenyl)piperazine **5** for series **A** or 1-(2-chloro-1-oxoethyl)-4-(3,4-dichlorophenyl)piperazine **6** or 1-(2-chloro-1-oxoethyl)-4-(m-trifluoromethylphenyl)piperazine **7** for series **B**. Compounds of series **A** were obtained with a yield of 32–40% and series **B** with a yield of 37–67%.

### 2.2. In Vitro Antiproliferative Activity

The cytotoxicity of new compounds was tested in the non-tumorigenic epithelial cell line (MCF10A) and breast adenocarcinoma cell line (MCF7) using the MTT method. The effect of new compounds on cancerous MCF7 and normal MCF10A cells alone and in combination with doxorubicin was investigated.

#### 2.2.1. Effect of New Compounds on MCF10A and MCF7 Cell Viability

**Figure 4:**
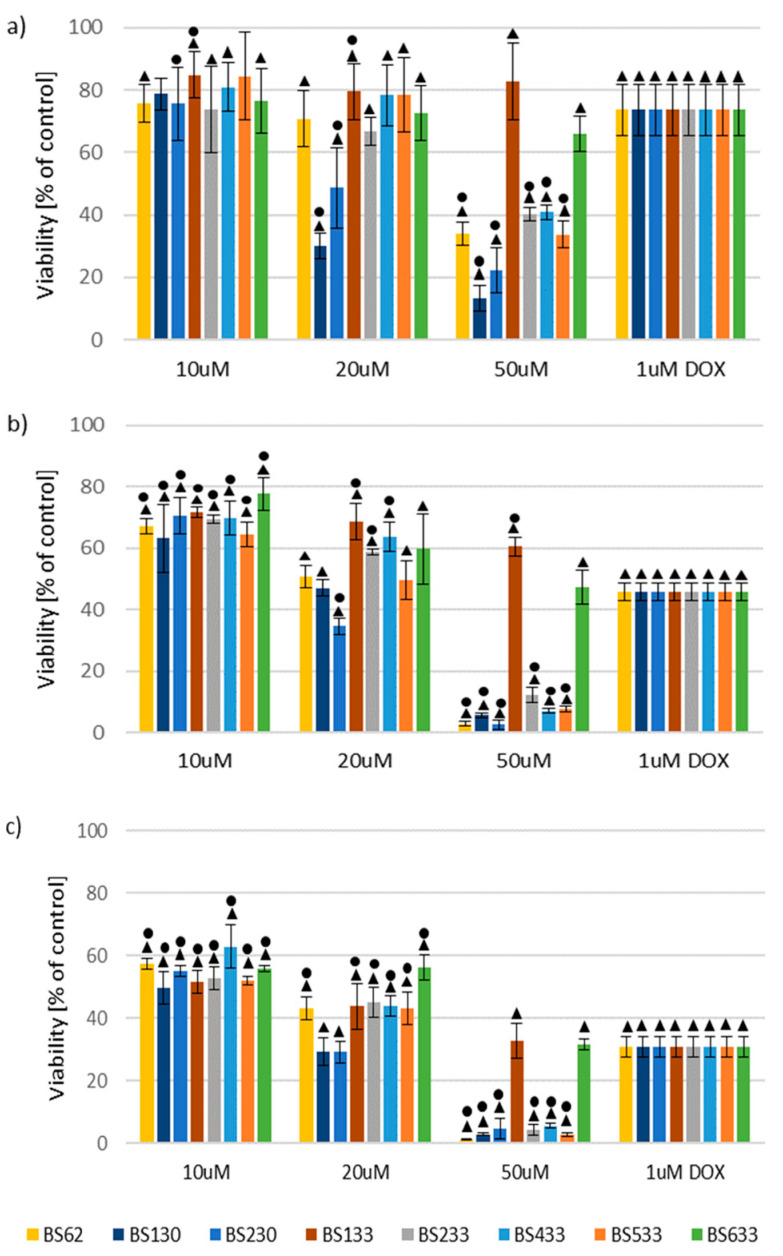
Viability of MCF10A cells treated with **BS** compounds for (**a**) 24 h, (**b**) 48 h, and (**c**) 72 h. The triangle indicates statistical significance compared to the non-treated cells. The dot indicates statistical significance compared to the cells treated with 1 µM DOX.

Firstly, all new compounds were tested at three different concentrations (10, 20, and 50 µM) to assess their cytotoxicity to non-tumorigenic and tumorigenic cell lines in three different time periods (24, 48, and 72 h). Doxorubicin at a concentration of 1 µM was the reference drug. The addition of studied compounds with increasing concentrations to the culture medium resulted in a decrease in the viability of MCF10A cells in all three time points (24, 48, and 72 h) (Figure 4). Exceptions are **BS130** and **BS533** compounds at concentrations of 10 µM, which after 24 h did not cause a significant decrease in cell viability compared to untreated cells. As you can see in Figure 4a, **BS130** and **BS533** are not marked with a dot or a triangle at a concentration of 10 µM. The triangle indicates statistical significance compared to the non-treated cells. The dot indicates statistical significance compared to the cells treated with 1 µM DOX.

**Figure 5:**
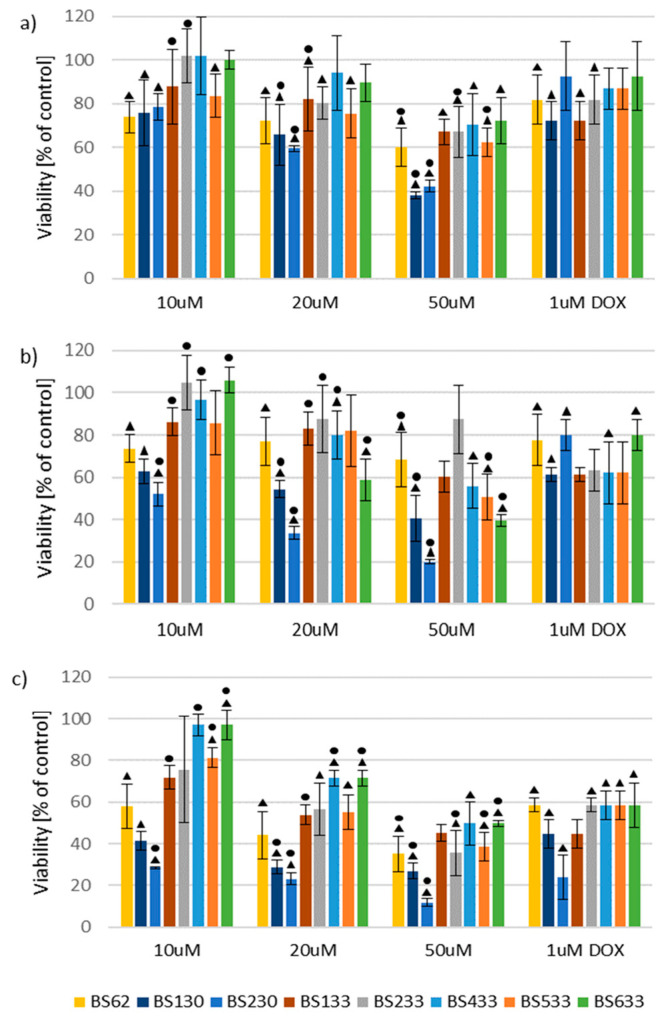
Viability of MCF7 cells treated with **BS** compounds for (**a**) 24 h, (**b**) 48 h, and (**c**) 72 h. The triangle indicates statistical significance compared to the non-treated cells. The dot indicates statistical significance compared to the cells treated with 1 µM DOX.

The addition of the tested compounds with increasing concentrations to the culture medium of the MCF7 cancer cells also mostly increased toxicity in all three time points (Figure 5). The exception was the **BS62** compound after 48 h of incubation, which was less toxic at concentrations of 20 µM than 10 µM (Figure 5b).

#### 2.2.2. Effect of New Compounds in Combination with Doxorubicin on MCF10A and MCF7 Cell Viability

All new compounds were tested also at three different concentrations (10, 20, and 50 µM) in combination with doxorubicin at two concentrations (1 and 5 µM), in three different time periods (24, 48, and 72 h).

##### Effect of New Compounds in Combination with Doxorubicin on MCF10A Cell Viability

**Figure 6:**
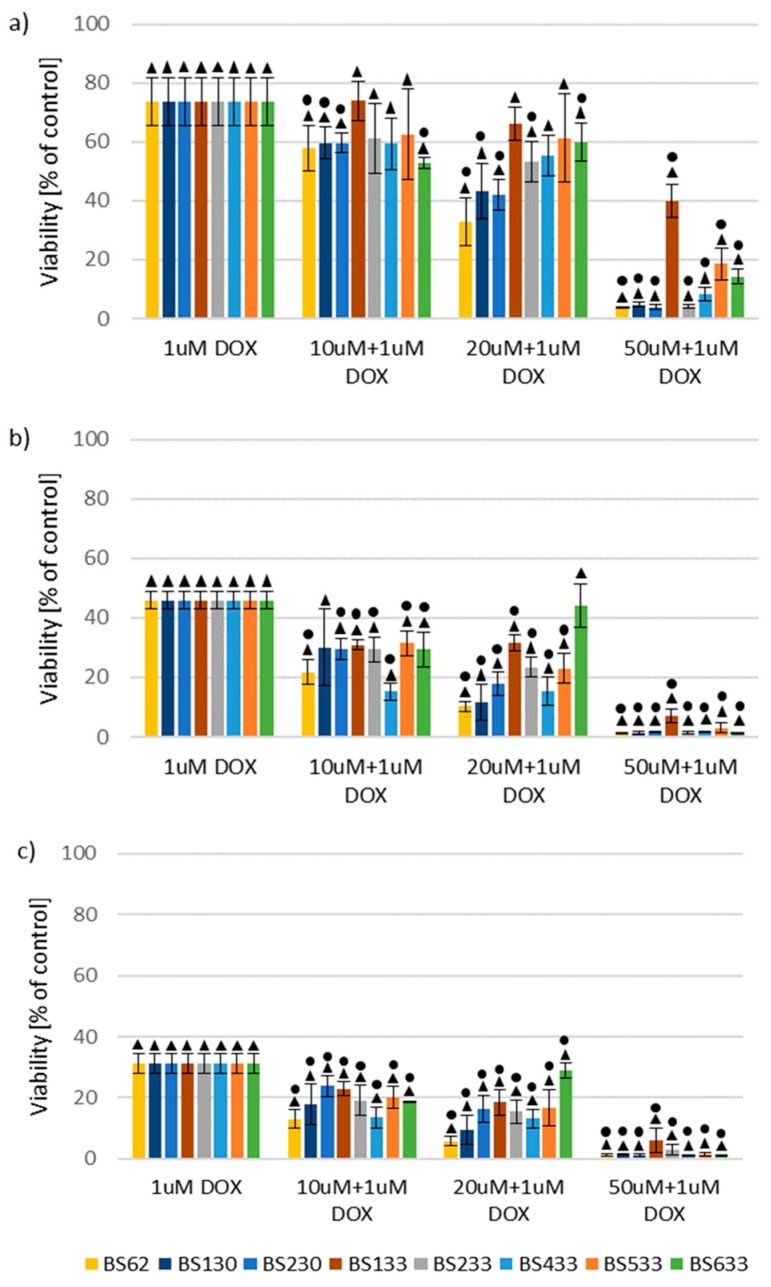
Viability of MCF10A cells treated with **BS** compounds in combination with DOX (1 µM) for (**a**) 24 h, (**b**) 48 h, and (**c**) 72 h. The triangle indicates statistical significance compared to the non-treated cells. The dot indicates statistical significance compared to the cells treated with 1 µM DOX.

The addition of the studied compounds in combination with doxorubicin at a concentration of 1 µM to the culture medium of the MCF10A cells did not significantly decrease the toxicity of DOX to MCF10A cells (Figure 6). For example, **BS133**, **BS433**, and **BS533** compounds at concentrations of 10 and 20 µM and **BS233** at a concentration of 10 µM in combination with DOX after 24 h did not significantly affect the decrease the viability of MCF10A cells compared to the cells treated with 1 µM DOX alone. The cell viability did not significantly decrease after 48 h either with the **BS130** compound at a concentration of 10 µM, as well as **BS633** at a concentration of 20 µM in combination with 1 µM DOX compared to the cells treated with 1 µM DOX alone.

The results of the addition of the studied compounds in combination with doxorubicin at a concentration of 5 µM to the culture medium of the MCF10A cells are presented in the Supplementary Materials (Figure S24). Compounds **BS133**, **BS233**, and **BS533** at concentrations of 10 and 20 µM after 24 h significantly decreased the toxicity of doxorubicin at concentrations of 5 µM. Higher viability after 24 h was also observed in cells treated with **BS230** at a concentration of 10 µM compared to cells treated with only 5 µM DOX. However, the addition of **BS62**, **BS130**, and **BS433** compounds at concentrations of 10 and 20 µM, **BS633** at concentrations of 10, 20, and 50 µM, **BS133** at concentration of 50 µM, and **BS230** at concentration of 20 µM after 24 h did not significantly affect the change in the MCF10A cell survival compared to the cells treated with 5 µM DOX alone. After 48 and 72 h, none of the tested compounds significantly decreased the toxicity of 5 µM DOX to the MCF10A cells compared to the cells treated with only 5 µM DOX.

##### Effect of New Compounds in Combination with Doxorubicin on MCF7 Cell Viability

**Figure 7:**
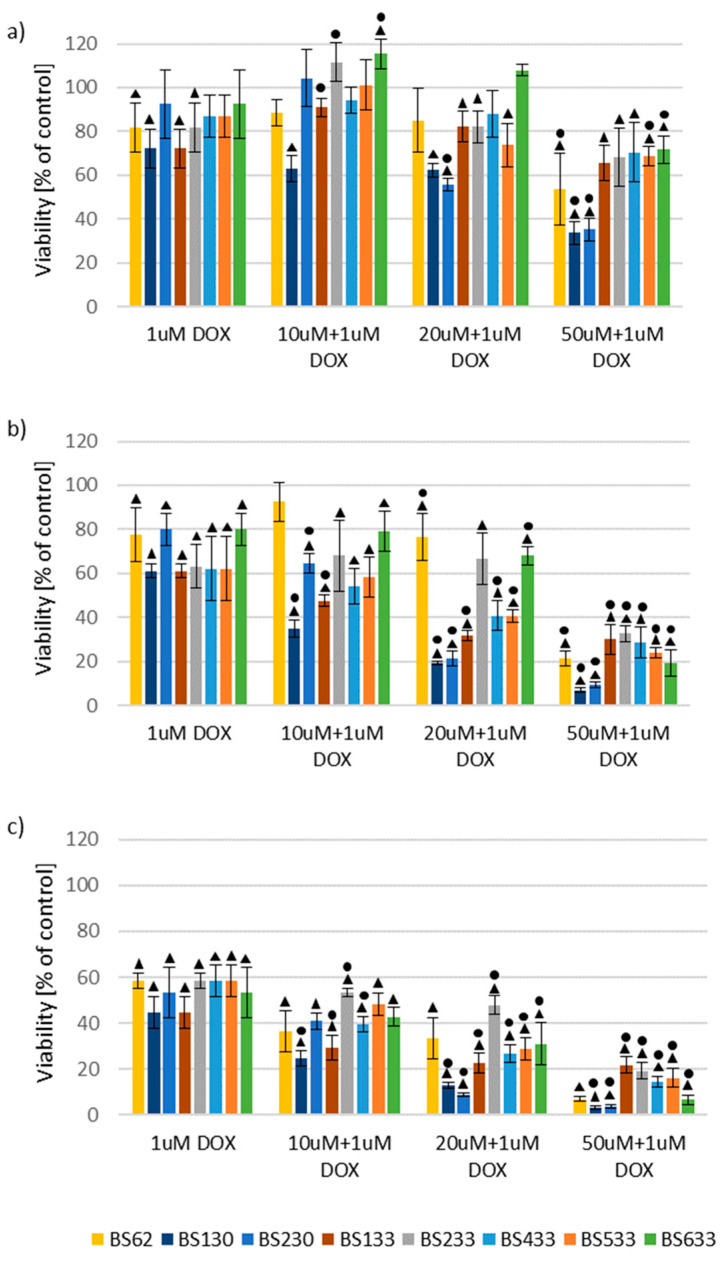
Viability of MCF7 cells treated with **BS** compounds in combination with DOX (1 µM) for (**a**) 24 h, (**b**) 48 h, and (**c**) 72 h. The triangle indicates statistical significance compared to the non-treated cells. The dot indicates statistical significance compared to the cells treated with 1 µM DOX.

After 24 h of treating MCF7 cells, the reinforcement of 1 µM DOX toxicity was caused by the addition of **BS533**, **BS130**, **BS633**, and **BS62** compounds at a concentration of 50 µM and **BS230** at concentrations of 20 and 50 µM (Figure 7). After 48 h, the addition of **BS133** and **BS130** caused reinforcement of toxicity at all concentrations. After 48 h, the toxicity of 1 µM DOX significantly reinforced the addition of **BS533** at a concentration of 50 µM and **BS433** at concentrations of 20 and 50 µM. After 72 h, the toxicity of 1 µM DOX significantly reinforced the addition of **BS433**, **BS133**, and **BS130** at all concentrations and **BS533** at a concentration of 50 µM.

The results of the addition of the studied compounds in combination with doxorubicin at a concentration of 5 µM to the culture medium of the MCF7 cells are presented in the Supplementary Materials (Figure S25). After 24 h of treating MCF7 cells, significantly lower viability was observed with the addition of **BS62, BS233, BS633**, and **BS130** compounds at a concentration of 50 µM and **BS230** at concentrations of 20 and 50 µM to 5 µM DOX. Similarly, after 48 h of treatment, significantly lower viability was observed with **BS633** and **BS230** compounds at concentrations of 20 and 50 µM, **BS130** at concentration of 50 µM, as well as after 72 h of treatment with **BS62** at concentration of 50 µM.

#### 2.2.3. Structure–Activity Relationship (SAR) Analysis

##### SAR Analysis in the Study of the Effect of New Compounds on MCF10A and MCF7 Cell Viability

As the results of the studies show, the highest cytotoxicity against both healthy (MCF10A) and cancer (MCF7) cell lines is demonstrated by compounds of the **B** series—**BS130**, **BS230**, and **BS62**. Series **A** compounds differ from series **B** compounds mainly in the type of substituent at the thiazine nitrogen atom. In series **A**, the phenylpiperazine substituent contains a fluorine atom at position 4 of the phenyl ring. In series **B**, the phenylpiperazine group contains two chlorine atoms (at positions 3 and 4) or a trifluoromethyl substituent at position 3. From the series **B**, the strongest effect was given by compounds **BS130** and **BS230**, which suggests that two chlorine atoms in the phenylpiperazine substituent are very important for the cytotoxic effect of the new 1,2-benzothiazine derivatives, stronger than in the case of the fluorine or trifluoromethyl substituent. The **BS230** compound, which, when compared to doxorubicin, shows a more favorable profile of action than **BS130**, in addition to two chlorine atoms in the phenylpiperazine, has an additional chlorine atom in the para position of the benzoyl substituent.

However, within the **A** series compounds, only subtle differences in cytotoxic activity can be seen, especially on the MCF7 cancer line. Series **A** compounds differ only in the type of substituent in the benzoyl group. The results of these studies show that this substituent does not have such a significant effect on the cytotoxic activity. However, the strongest cytotoxic activity on MCF10A cells in this series was demonstrated by the chlorine-substituted compound (**BS233**), while on MCF7 cells it was the methyl-substituted compound (**BS533)**. A slightly weaker effect was observed for compounds with a methoxy substituent (**BS433**). The compound with the weakest cytotoxic activity on both cell lines, but only slightly, was the compound with a fluorine substituent (**BS633**). Additionally, on the MCF10A healthy cell line, the **BS133** compound without a substituent in the benzoyl group was distinguished by very low cytotoxicity. The activity of compounds towards a healthy cell line (MCF10A) can be ranked as follows:

**BS130** ≥ **BS230** > **BS62** ≥ **BS233** ≥ **BS433** ≥ **BS533** > **BS133** ≥ **BS633**

whereas in terms of the cancer cell line (MCF7) we can arrange it as follows:

**BS130** (after 24 h) ≈ **BS230** (after 48 and 72 h) > **BS62** (after 24 and 72 h) ≈ **BS533** ≥ **BS233** (after 24 and 72 h) > **BS133** > **BS433** (after 24 and 72 h) ≈ **BS633** (after 24 and 72 h)

At concentrations of 10 and 20 µM, most compounds showed lower cytotoxicity than doxorubicin (except **BS130** and **BS230** at concentrations 20 µM). At a concentration of 50 µM, most compounds showed higher cytotoxicity than doxorubicin (except **BS133** and **BS633**).

##### SAR Analysis in the Study of the Effect of New Compounds in Combination with Doxorubicin on MCF10A and MCF7 Cells Viability

On the healthy MCF10A cell line, all tested compounds in combination with doxorubicin at a concentration of 1 µM reduced cell viability at all applied concentrations (10, 20, and 50 µM) and time intervals (24, 48, and 72 h) compared to doxorubicin alone. There was no significant difference between the effects of the series **A** and series **B** compounds as in the previous study. The lowest cytotoxicity after 24 h was observed for series **A** compounds without a substituent in the benzoyl group (**BS133**) and after 48 and 72 h for series **A** compounds with a fluorine substituent (**BS633**), which is consistent with the results of the previous study. Although the cytotoxic activity of the remaining compounds was comparable, the series **B** compound with trifluoromethyl substituent (**BS62**) was slightly more pronounced. 

The synergistic effect of the tested compounds and DOX at a concentration of 5 µM on the MCF10A cell line was diverse. After 24 h of incubation, some of the tested compounds increased the cytotoxicity of DOX (e.g., **BS62**, **BS130**) and some of the compounds reduced it (e.g., **BS133**, **BS233**, **BS533**) in a dose-dependent manner. However, after 48 and 72 h of incubation, the effects of all compounds were equal and were at the DOX toxicity level.

In the case of the MCF7 cancer cell line at a dose of 10 µM and 20 µM of the compound with the addition of 1 µM DOX, some compounds enhanced DOX cytotoxicity (e.g., **BS130**, **BS230**), while others showed reduced toxicity or there was no significant difference (e.g., **BS133**, **BS233**, **BS633**). However, at a dose of 50 µM, all tested compounds in combination with DOX showed greater cytotoxicity than doxorubicin alone at all three time intervals.

A similarly differentiated effect was seen in the study of compounds in combination with DOX at a concentration of 5 µM. The least cytotoxic was series **A** compound without a substituent in the benzoyl group (**BS133**), while the remaining compounds showed similar cytotoxicity dependent on the dose and time of action. 

In these studies, once again, series **B** compounds **BS130** and **BS230** enhanced the cytotoxicity of doxorubicin, which showed that two chlorine atoms in the phenylpiperazine substituent are very important for the cytotoxic effect of the new 1,2-benzothiazine derivatives. The activity of compounds in combination with doxorubicin (at a concentration of 1 µM) towards a healthy cell line (MCF10A) can be ranked as follows:

**BS62** > **BS130** ≈ **BS230** ≈ **BS433** > **BS233** ≈ **BS533** ≈ **BS633** > **BS133**

whereas in terms of the cancer cell line (MCF7) we can arrange it as follows:

**BS130** > **BS230** > **BS133** > **BS433** ≈ **BS533** > **BS62** > **BS233** > **BS633**

#### 2.2.4. Therapeutic Potential of the Tested Compounds

Based on the analysis of the obtained results, compounds with potential therapeutic effects were identified. The toxicity of the tested compounds was analyzed compared to the commonly used anticancer drug doxorubicin, focusing on the following:

- Decreased viability of MCF7 cells while maintaining toxicity similar to DOX towards MCF10A cells;
- Decreased toxicity towards MCF10A cells while maintaining toxicity similar to DOX towards MCF7 cells;
- Decreased viability of MCF7 cells while simultaneously reducing toxicity towards MCF10A cells.

**Figure 8:**
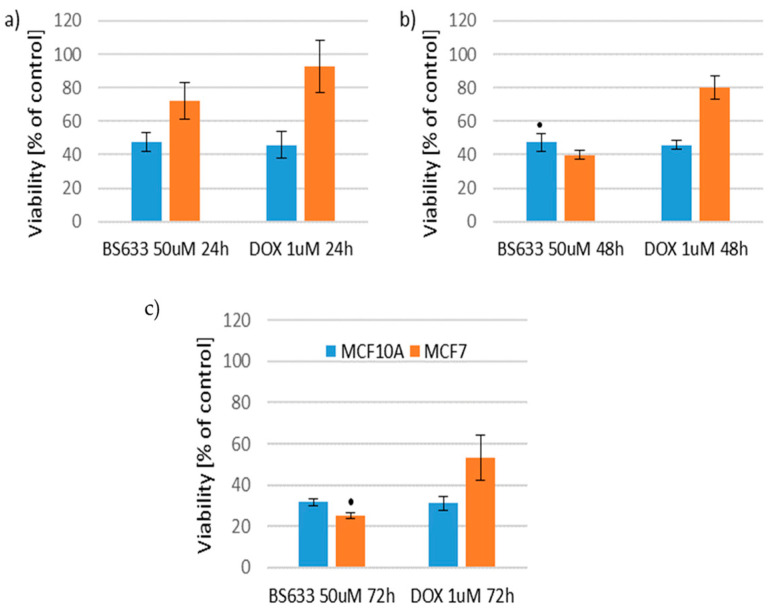
Viability of MCF10A and MCF7 cells treated with **BS633** (50 µM) compound compared to corresponding cells treated with 1 µM DOX for (**a**) 24 h, (**b**) 48 h, and (**c**) 72 h. The dot indicates statistical significance compared to the respective cells treated with 1 µM DOX.

The compound **BS633** at a concentration of 50 µM was classified into the first group, which after 24 h of MCF10A and MCF7 cell treatment showed no significant differences compared to treating both lines with 1 µM DOX, whereas after 48 and 72 h, it significantly impacted the reduction of MCF7 cell viability by 40% and 28%, respectively, compared to cells treated with 1 µM DOX, while not significantly affecting the reduction of MCF10A cell viability compared to cells treated with 1 µM DOX (Figure 8).

**Figure 9:**
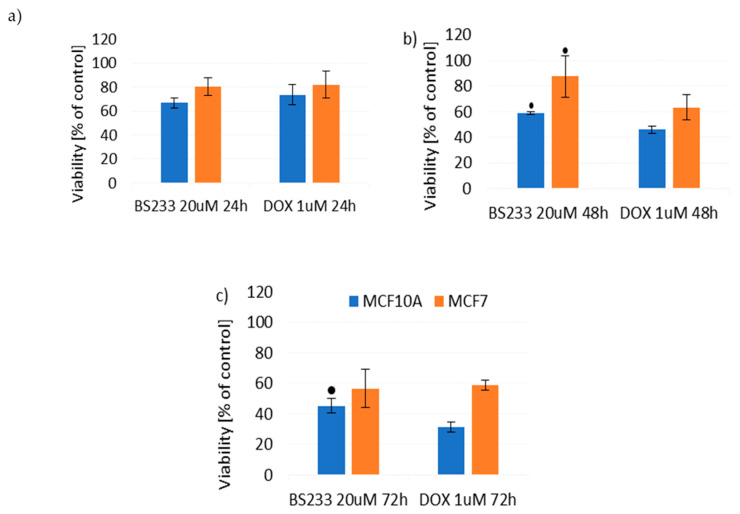
Viability of MCF10A and MCF7 cells treated with **BS233** (20 µM) compound compared to corresponding cells treated with 1 µM DOX for (**a**) 24 h, (**b**) 48 h, and (**c**) 72 h. The dot indicates statistical significance compared to the respective cells treated with 1µM DOX.

The second group included compounds **BS233**, **BS62**, and **BS633** at a concentration of 20 µM, which for 24 h and 48 h did not show significantly beneficial differences in the viability of treated cells compared to treating cells with 1 µM DOX, whereas after 72 h of treatment, they significantly improved the viability of MCF10A cells by causing lower toxicity of 15% and 25%, respectively, compared to cells treated with 1 µM DOX, while maintaining comparable toxicity to 1 µM DOX towards MCF7 cells (Figure 9 and Figures S26 and S27 in Supplementary Materials). 

**Figure 10:**
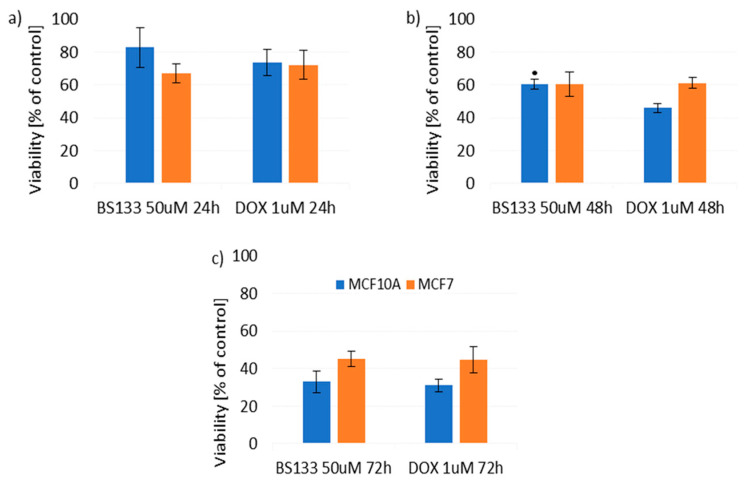
Viability of the MCF10A and MCF7 cells treated with **BS133** (50 µM) compound compared to corresponding cells treated with 1 µM DOX for (**a**) 24 h, (**b**) 48 h, and (**c**) 72 h. The dot indicates statistical significance compared to the respective cells treated with 1 µM DOX.

Also, compounds **BS133** at a concentration of 50 µM and **BS533** at a concentration of 10 µM, which only after 48 h of action showed a significantly beneficial effect towards MCF10A cells, maintaining around 15% more cells in the population compared to the population treated with 1 µM DOX, without compromising the toxicity of 1 µM DOX towards MCF7 cells (Figure 10 and Figure S28 in Supplementary Materials).

The compound **BS62** at a concentration of 10 µM after 48 h and 72 h of treating MCF10A cells was significantly less toxic than 1 µM DOX (by 21% and 26%, respectively) and equally effective against MCF7 cells as 1 µM DOX (Figure S29 in Supplementary Materials). Similarly, the compound **BS130** at a concentration of 10 µM at all three time points resulted in significantly lower toxicity towards MCF10A cells compared to cells treated with 1 µM DOX (5% for 24 h and 18% for 48 and 72 h), while maintaining efficacy against MCF7 cells comparable to 1 µM DOX (Figure S30 in Supplementary Materials). In the context of the three analyzed situations, the compound **BS433** did not show significantly beneficial differences towards the MCF10A or MCF7 cells compared to cells treated with 1 µM DOX at any time point. 

**Figure 11:**
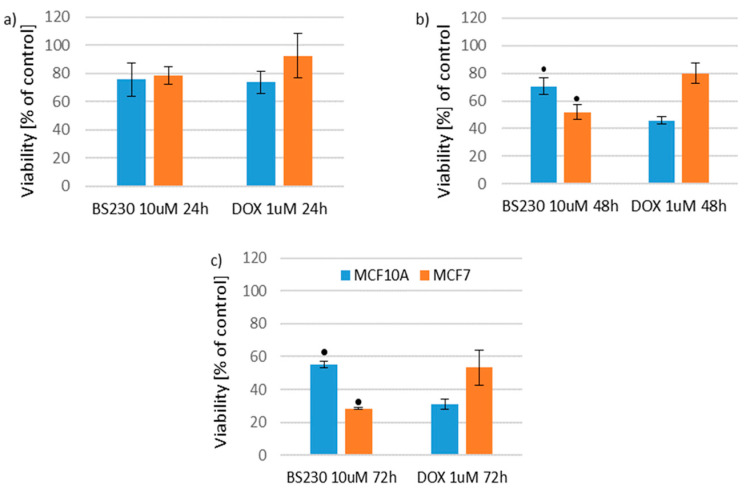
Viability of the MCF10A and MCF7 cells treated with the **BS230** (10 µM) compound compared to the corresponding cells treated with 1 µM DOX for (**a**) 24 h, (**b**) 48 h, and (**c**) 72 h. The dot indicates statistical significance compared to the respective cells treated with 1 µM DOX.

The third group included the compound **BS230** at a concentration of 10 µM. After 24 h, it did not lead to significant differences in the viability of both cell lines compared to cells treated with 1 µM DOX, whereas after 48 and 72 h, it significantly reduced the viability of MCF7 cells by 29% and 25%, respectively, compared to cells treated with 1 µM DOX, with a simultaneous decrease in toxicity towards MCF10A cells by 24% at both time points compared to cells treated with 1 µM DOX (Figure 11).

This analysis shows that the compound with the most beneficial therapeutic effect from this group of new compounds is **BS230** because it decreased viability of the cancer cells (MCF7) while simultaneously reducing toxicity towards the healthy cells (MCF10A) in comparison to DOX.

### 2.3. Fluorescence Spectroscopic Study of Binding to DNA

One of the modes of anticancer DNA-targeted drugs is their interaction with the minor groove of double-stranded nucleic acids. Therefore, in order to analyze the binding of all compounds to ct-DNA, fluorescence measurements were performed. These were performed in the presence of constant concentrations of ct-DNA and Hoechst 33342, which is a bis-benzimide derivative that binds to the minor groove of double-stranded DNA. It is known that Hoechst 3334 bounded to DNA exhibits maximum fluorescence at λ_em_ = 458 nm when excited to λ_exc_ = 349 nm. Such a procedure has been successfully used in previous studies [24,25]. 

**Figure 12:**
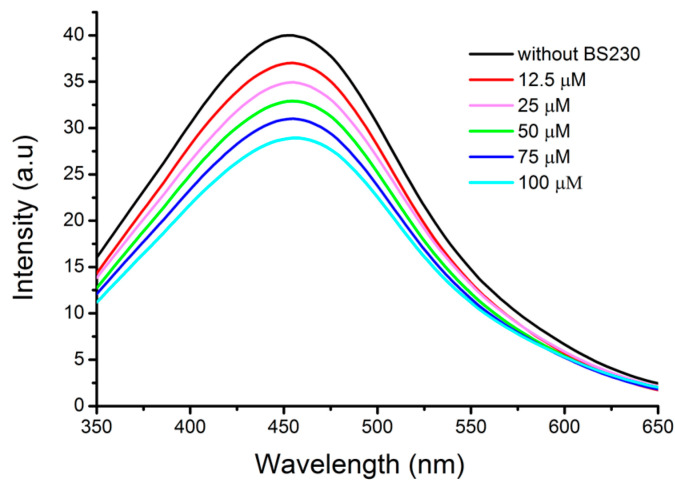
Emission spectra of ct-DNA/Hoechst complex ([ct-DNA] = 0.19 µM and [Hoechst 33342] = 15 µM), λ_ex_ = 349, λ_em_ = 458 nm, upon addition of the increasing amounts (0–100 µM) of **BS230**.

**Figure 13:**
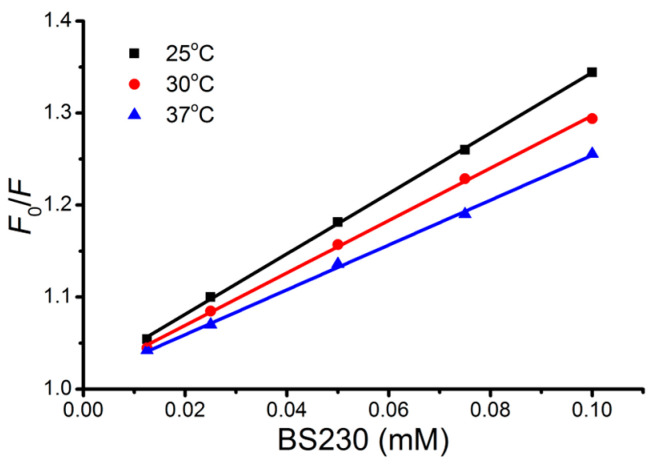
*Stern–Volmer* plot for the interaction of **BS230** with ct-DNA/Hoechst 33342 at three different temperatures.

In Figure 12 and Figure 13, the results obtained for the most active against cancer cell line and non-toxic for normal human breast cell line compound **BS230** are shown.

Due to the increasing amount of **BS230**, a clear decrease in Hoechst 33342-DNA complex emission intensity is noticeable. Additionally, no shift of fluorescence maximum emission has taken place. Consequently, we can assume the presence of interactions between **BS230** and ct-DNA and gradual replacement of stain in the minor groove of nucleic acid.

We also analyzed the quenching properties of designed compounds taking into account the *Stern–Volmer* equation, and the k_q_ value was obtained as a slope from the plot of F_0_/F versus [Q]. The linear trend, that can be seen in Figure 13, suggests one major binding mode due to the single donor quenching system. The K_sv_ values obtained for all compounds are presented in Table 1.

Their magnitude varies from 1.35 × 10^3^ (M^−1^) for **BS133** to 3.411 × 10^3^ (M^−1^) for **BS633**, respectively. As can be seen, the *Stern–Volmer* quenching constants were determined to be lower than the well-known classical constants, which confirms the groove-binding mechanism of action. If we consider the results for **BS230** presented in Figure 13, we can see that the values of K_SV_ decreased with the increasing temperature, which indicated that the fluorescence quenching is static [25]. In order to visualize the phenomena described above, we also performed molecular docking. The obtained results are presented in Section 2.4.

### 2.4. Molecular Docking Studies

**Figure 14:**
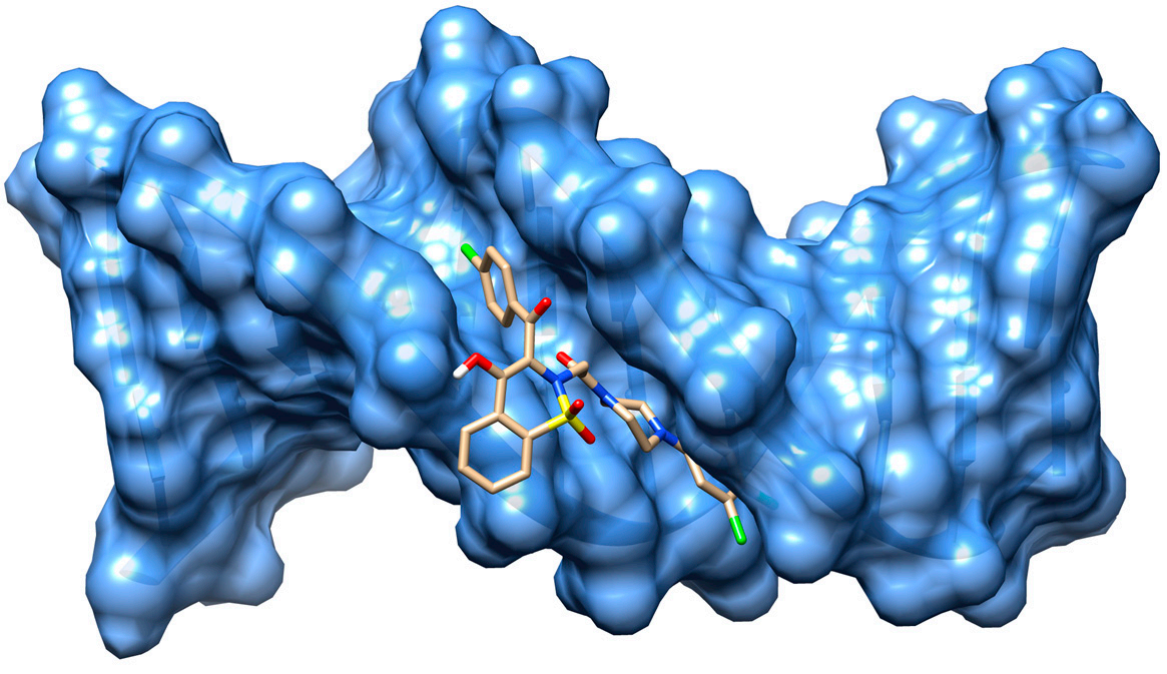
The best docking pose of **BS230** compound in DNA.

**Figure 15:**
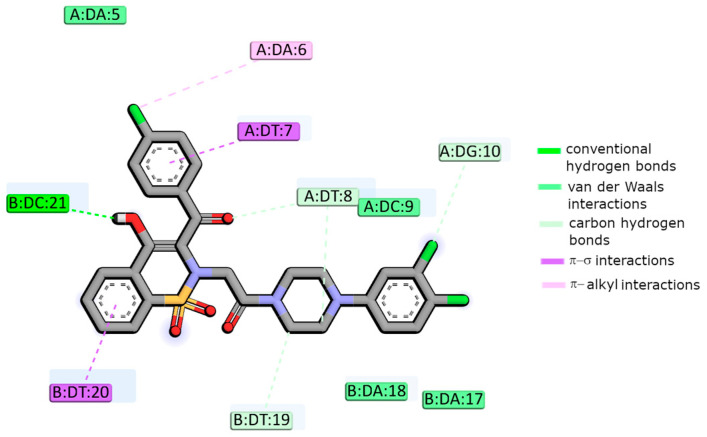
The intermolecular interactions of **BS230** compound and DNA.

As experimental data suggest, all designed compounds may interact with DNA in a minor groove. Therefore, we also performed their molecular docking to DNA to show possible modes of binding. The data reveal that all compounds interact with DNA to form stable complexes with a negative energy scoring function. Results obtained for **BS230** are presented in Figure 14 and Figure 15.

As presented, the conformation of the designed compound allows for interactions in the major or minor groove of DNA. In this case, one hydrogen bond is formed between adenine DC21 and the hydroxyl group of 1,2-benzothiazine. The other three carbon–hydrogen bonds are also possible with DT8, DG10, and DT19. The system is also stabilized by a series of van der Waals forces. For example, DA5, DA9, DA17, and DA18 are involved in that type of interaction. Additionally, π-type interactions are the origins of complex stabilization. In terms of ∆G_binding_, compound **BS230** binds most strongly to DNA (−63.2 kJ/mol). However, as you can see in Table 2, the differences in free energies of binding are not significant. The detailed data obtained for **BS230** and remaining derivatives are shown in the Supplementary Materials.

According to an experimental study, designed compounds exhibit cytotoxic activity against the breast adenocarcinoma cell line (MCF7). One of the recognized molecular targets for drugs used in the treatment of breast cancer are topoisomerases. For example, it has been proven that DOX induces DNA double helix cleavage through Topo II. Therefore, we proposed the binding mode of designed compounds to Topo IIα based on the results of molecular docking. Due to the lack of DOX-Topo IIα complex crystals in the database, to validate our docking procedure, we used proteins complexed with etoposide (ETO), another Topo II inhibitor.

**Figure 16:**
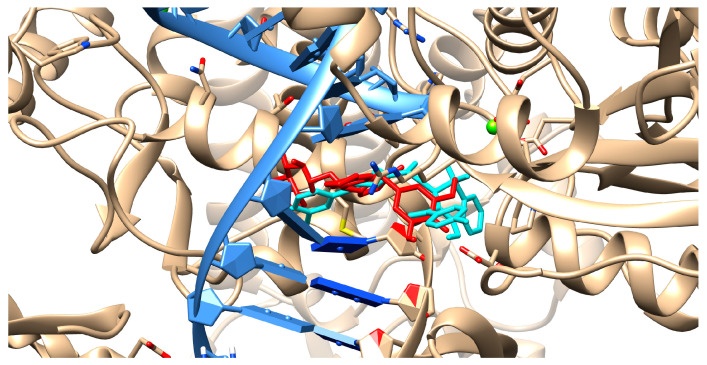
The binding mode of **BS230** (cyan) and etoposide (red) in the active site of Topo IIα. In the 3D representation, the protein chain is marked in gold and the DNA structure is blue.

**Figure 17:**
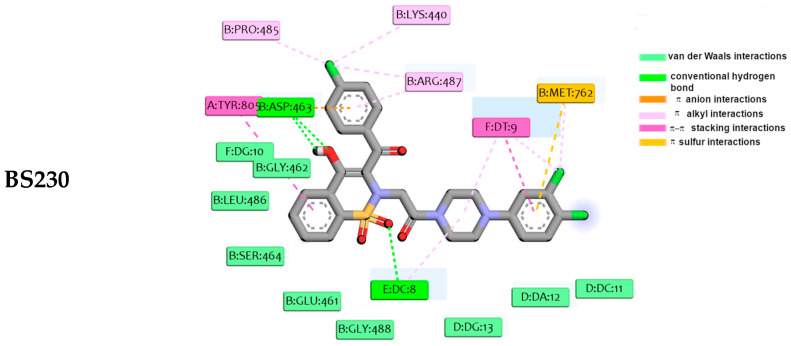

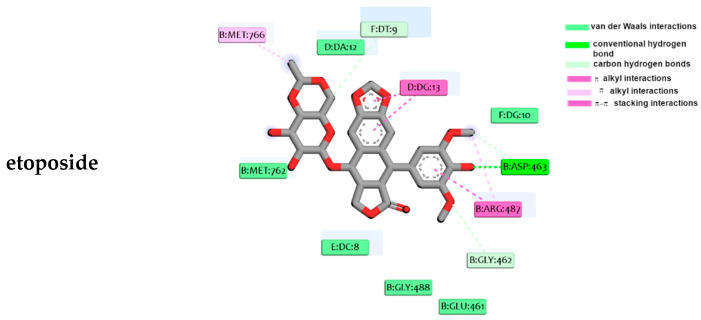
Intermolecular interactions in the active site of Topo IIα (2D representation) of **BS230** and etoposide.

According to the results, all considered compounds can bind in the active center of Topo IIα and can partially interact with DNA. The most potent inhibitor, taking into account the scoring function including free energy of binding, is compound **BS230**. The estimated ∆G_binding_ in this case is equal to −66.6 kJ/mol, while for the reference drug it is −44.6 kJ/mol. It should be mentioned that for the other compounds it varies in the range from −63.0 kJ/mol to −65.6 kJ/mol. In Figure 16 and Figure 17, we present the result obtained for the most toxic compounds towards the adenocarcinoma cell line—**BS230**. As can be observed in Figure 16 and Figure 17, compound **BS230** binds in a similar manner as the reference drug, etoposide.

The 3,4-dichlorophenylpiperazine moiety, similar to the aglycone part of etoposide, is involved in drug-DNA interactions with DC8, DT9, DC11, DC12, and DG13. Additionally, the 3,4-dichlorophenyl ring participates in drug–protein π-sulfur interactions with Met762. The planar polyaromatic system of 1,2-benzothiazine is localized in the cavity created by Glu461, Gly462, Asp463, Leu486 and Gly488. Similar binding mode exhibits the podophyllotoxin moiety of the reference drug. Due to the presence of aromatic rings in the structure of designed inhibitors, π-π interactions with Tyr805 and DT9 are also present.

Results obtained for the other compounds are presented in Figures S17–S23 in the Supplementary Materials. Most of the derivatives have a very similar interaction pattern. The phenylpiperazine part of the molecules slides between nucleic acid bases and is involved in π-π stacking interactions mainly with the DT9 and DA12 nucleobases. Additionally, in many cases, extra π-sulfur interactions with Met762 are present. The hydrogen bonds between the 1,2-benzothiazine moiety and DC8 and DT9 nucleobases, and Asp463 and Gly488 amino acid residues are possible.

## 3. Summary

In a three-step synthesis, two series of 1,2-benzothiazine derivatives were obtained, differing especially in the substituent at the thiazine nitrogen atom. The cytotoxicity of the new derivatives was examined by the MTT test on the non-tumorigenic epithelial cell line (MCF10A). All compounds at concentrations of 10 µM in all three time intervals (24, 48, and 72 h) showed significantly lower toxicity than doxorubicin—the reference drug—at concentrations of 1 µM. Another study was carried out on a breast adenocarcinoma cell line (MCF7) to confirm the anticancer activity of the new compounds. The compounds showed varied activity. The most cytotoxic compounds, even more than doxorubicin, were **BS130** and **BS230**, i.e., compounds containing a 1-(3,4-dichlorophenyl)piperazine substituent in their structure. These results show that the phenylpiperazine derivative with two chlorine atoms in the phenyl ring is definitely preferred for cytotoxic activity in this group of compounds.

The next study aimed to determine whether the new compounds would act synergistically with doxorubicin and enhance its cytotoxic effect. On the healthy (MCF10A) cell line, all tested compounds in combination with doxorubicin reduced cell viability, i.e., enhanced doxorubicin cytotoxicity at all applied concentrations (10, 20, and 50 µM) and time intervals (24, 48, and 72 h). However, this synergistic effect was not as pronounced in the MCF7 cancer cell line. At doses of 10 µM and 20 µM of the compound (with the addition of 1 µM DOX), some compounds enhanced DOX cytotoxicity (e.g., **BS130**, **BS230**, and **BS533**), while others showed reduced toxicity or there was no significant difference (e.g., **BS133**, **BS233**, and **BS633**). However, at a dose of 50 µM, all tested compounds in combination with DOX showed higher cytotoxicity than doxorubicin alone at all three time intervals. In this study, once again, compounds **BS130** and **BS230** enhanced the cytotoxicity of doxorubicin, which showed that they can act synergistically with DOX.

As a result of comparing the effects of the tested compounds on healthy and cancer cells, the compounds were divided into three groups: compounds more potently cytotoxic than DOX towards cancer cells, but just as toxic as DOX towards healthy cells; compounds cytotoxic towards cancer cells to a similar extent as DOX, but with lower toxicity towards healthy cells; and compounds more potently cytotoxic than DOX towards cancer cells and lower toxicity towards healthy cells. The latter group, which includes compound **BS230**, shows the best therapeutic potential as a new safer anticancer drug.

One of the many molecular targets for anticancer drugs is DNA; therefore, studying the binding of the compound to ct-DNA using spectroscopic methods provides additional information useful in determining the mechanism of action of the new compounds. The results of this study showed that all the compounds tested interact with ct-DNA in a similar way, proportional to the concentration of the compound. This study confirmed also the groove-binding mechanism of action of the new compounds. To visualize this phenomenon, we also performed a molecular docking study. The data revealed that all compounds interact with DNA to form stable complexes with a negative energy scoring function, and all considered compounds can bind in the active center of topoisomerase IIα.

There are many experimental data concerning the anticancer activity of piperazine and benzothiazine derivatives [26]. One possible mechanism of action is the inhibition of topoisomerases. Topoisomerase poisons are able to block off broken DNA ends, rejoining what causes DNA double-strand breakage by replicating broken DNA by cells. Manasa et al. designed and analyzed a series of new piperazine derivatives in terms of their anticancer activity against a panel of human cancer cell lines. It was shown that the designed compounds exhibit cytotoxic activity and induce apoptosis of cancer cells. Additionally, sulfonyl piperazine compounds exhibit inhibitory activity towards Topo II. The results of spectroscopic and molecular docking studies indicated their ability to bind to topoisomerase and DNA. Our results are in good agreement with previous experimental data. Similarly, our compounds can form hydrogen bonds with Asp amino acid residues. The benzothiazine and phenylpiperazine rings are responsible for π-type interactions with aromatic amino acids in the nucleic acids binding domain.

It should be mentioned here that it is difficult to determine the mode of compounds binding to duplex DNA. As known, there are two possibilities: the interaction with the minor groove or the intercalation process. However, our experimental and theoretical results support binding to the minor groove. Previous findings suggested that compounds with the planar ring moieties could also be an intercalator, and mechanisms of binding could be mixed.

## 4. Materials and Methods

### 4.1. Chemistry

The melting points were recorded using MEL-TEMP capillary melting point apparatus and were uncorrected. ^1^H and ^13^C NMR spectra were measured on a Bruker NMR AVANCE III™ 600 MHz or 300 MHz spectrometer (Billerica, MA, USA) using TMS as an internal reference. The samples were prepared by dissolving in CDCl_3_. Chemical shift (δ) values are given in parts per million (ppm). Splitting patterns are designated as follows: s, singlet; brs, broad singlet; d, doublet; t, triplet; q, quartet; m, multiplet. FT-IR spectra were recorded on a Perkin-Elmer Spectrum Two UATR FT-IR spectrometer (Waltham, MA, USA). Accurate masses were obtained on a Bruker Daltonics micrOTOF-Q mass spectrometer in a positive ion mode with flow injection electron spray ionization (ESI). The elemental analyses (C, H, N) were performed by a Carlo Erba NA 1500 analyzer (Thermo Fisher Scientific, Waltham, MA, USA) and were within ±0.4% of the theoretical value. The samples were applied as solids. The progress of reactions and the purity of the prepared compounds were monitored by thin-layer chromatography (TLC) using Fluka-precoated aluminum plate silica gel with fluorescent indicator 254 nm (Sigma-Aldrich, Darmstadt, Germany). Reagents and solvents were purchased from commercial suppliers and used as received.

***General procedure for the preparation of series A and series B compounds ***

Synthesis and experimental data of compounds **3** and **4** were previously reported [23,27,28], and compounds **5**, **6**, and **7** were also reported [29,30].

***Synthesis and properties of series A compounds ***

To the mixture of compound **4** (5 mmol) in 20 mL of anhydrous ethanol with sodium ethoxide (2.3%, 5 mL) was added 1-(2-chloro-1-oxoethyl)-4-(*p*-fluorophenyl)piperazine **5** (1.28 g, 5 mmol) and refluxed with stirring for 8–10 h. When the reaction ended, which was controlled on TLC plates, ethanol was distilled off, the residue was treated with 50 mL of chloroform, and insoluble materials were filtered off. The filtrate was then evaporated, and the residue was purified by crystallization from ethanol to give desirable products with 32–40% yields. 

*3-Benzoyl-2-{2-[4-(p-fluorophenyl)-1-piperazinyl]-2-oxoethyl}-4-hydroxy-2H-1,2-benzothiazine 1,1-dioxide* (**BS133**). Light brown crystals, 39.10% yield, mp 143–145 °C; FT-IR (cm^−1^): 1663, 1591 (C=O), 1332, 1178 (SO_2_). ^1^H NMR (300 MHz, CDCl_3_) *δ* (ppm): 2.92 (m, 4H of piperazine), 3.29 (brs, 4H of piperazine), 3.69–3.71 (m, 2H, CH_2_-C=O), 6.89–8.26 (m, 13H, ArH), 15.60 (brs, 1H, OH*_enolic_*). ^13^C NMR (300 MHz, CDCl_3_) *δ* (ppm): 188.52, 171.69, 164.29, 138.50, 135.31, 133.28, 132.70, 132.60, 129.46, 129.00, 128.69, 127.73, 122.37, 119.10, 116.21, 116.07, 115.77, 58.41, 52.08, 50.82, 44.71, 41.24, 18.42. HRMS (ESI) calculated for C_27_H_24_FN_3_O_5_S [M + H]^+^ 522.1493; found the following: 522.1495. Analysis calculated for C_27_H_24_FN_3_O_5_S (521.56); C, 62.18; H, 4.64; N, 8.06; found the following: C, 62.10; H, 4.70; N, 8.10.

*3-(4-Chlorobenzoyl)-2-{2-[4-(p-fluorophenyl)-1-piperazinyl]-2-oxoethyl}-4-hydroxy-2H-1,2-benzothiazine 1,1-dioxide* (**BS233**). Light brown crystals, 35.37% yield, mp 130–134 °C; FT-IR (cm^−1^): 1668, 1589 (C=O), 1335, 1164 (SO_2_). ^1^H NMR (300 MHz, CDCl_3_) *δ* (ppm): 2.88–2.96 (m, 4H of piperazine), 3.37 (brs, 4H of piperazine), 3.68–3.72 (m, 2H, CH_2_-C=O), 6.96–8.26 (m, 12H, ArH), 15.47 (brs, 1H, OH*_enolic_*). ^13^C NMR (300 MHz, CDCl_3_) *δ* (ppm): 187.04, 171.77, 164.29, 139.11, 138.57, 133.62, 133.37, 132.65, 130.55, 129.38, 129.05, 127.76, 122.37, 119.30, 116.17, 115.88, 58.43, 52.00, 51.03, 44.67, 41.19, 18.42. HRMS (ESI) calculated for C_27_H_23_ClFN_3_O_5_S [M + H]^+^ 556.1104; found the following: 556.1113. Analysis calculated for C_27_H_23_ClFN_3_O_5_S (556.00); C, 58.32; H, 4.17; N, 7.56; found the following: C, 58.40; H, 4.20; N, 7.60.

*3-(4-Methoxybenzoyl)-2-{2-[4-(p-fluorophenyl)-1-piperazinyl]-2-oxoethyl}-4-hydroxy-2H-1,2-benzothiazine 1,1-dioxide* (**BS433).** Yellow powder, 32.12% yield, mp 110–113 °C; FT-IR (cm^−1^): 1666, 1593 (C=O), 1337, 1162 (SO_2_). ^1^H NMR (300 MHz, CDCl_3_) *δ* (ppm): 2.86–2.94 (m, 4H of piperazine), 3.34 (brs, 4H of piperazine), 3.70 (m, 1H, H_ax_ from CH_2_-C=O), 3.88 (s, 3H, OCH_3_), 4.20 (m, 1H, H_eq_ from CH_2_-C=O), 6.89–8.25 (m, 12H, ArH), 15.68 (brs, 1H, OH*_enolic_*). ^13^C NMR (300 MHz, CDCl_3_) *δ* (ppm): 187.80, 170.49, 164.42, 163.31, 138.50, 133.01, 132.47, 131.43, 129.57, 127.80, 127.59, 122.19, 119.11, 116.07, 115.85, 115.78, 114.06, 55.53, 51.83, 50.86, 44.75, 41.25. HRMS (ESI) calculated for C_28_H_26_FN_3_O_6_S [M + H]^+^ 552.1599; found the following: 552.1589. Analysis calculated for C_28_H_26_FN_3_O_6_S (551.58); C, 60.97; H, 4.75; N, 7.62; found the following: C, 60.00; H, 4.70; N, 7.60.

*3-(4-Methylbenzoyl)-2-{2-[4-(p-fluorophenyl)-1-piperazinyl]-2-oxoethyl}-4-hydroxy-2H-1,2-benzothiazine 1,1-dioxide* (**BS533**). Yellow crystals, 40.00% yield, mp 158–160 °C; FT-IR (cm^−1^): 1668, 1607 (C=O), 1335, 1160 (SO_2_). ^1^H NMR (300 MHz, CDCl_3_) *δ* (ppm): 2.44 (s, 3H, CH_3_), 2.87–2.96 (m, 4H of piperazine), 3.35 (brs, 4H of piperazine), 3.70–3.72 (m, 2H, CH_2_-C=O), 6.95–8.24 (m, 12H, ArH), 15.65 (brs, 1H, OH*_enolic_*). ^13^C NMR (300 MHz, CDCl_3_) *δ* (ppm): 188.46, 171.24, 164.42, 145.83, 143.63, 138.56, 137.88, 133.13, 132.59, 132.52, 131.41, 129.54, 129.42, 129.11, 127.66, 122.24, 119.45, 118.11, 116.17, 116.08, 115.88, 115.01, 51.92, 51.38, 50.85, 50.12, 44.56, 41.13, 21.79. HRMS (ESI) calculated for C_28_H_26_FN_3_O_5_S [M + H]^+^ 536.1650; found the following: 536.1627. Analysis calculated for C_28_H_26_FN_3_O_5_S (535.59); C, 62.79; H, 4.89; N, 7.85; found the following: C, 62.60; H, 4.90; N, 7.80.

*3-(4-Fluorobenzoyl)-2-{2-[4-(p-fluorophenyl)-1-piperazinyl]-2-oxoethyl}-4-hydroxy-2H-1,2-benzothiazine 1,1-dioxide* (**BS633**). Orange powder, 35.40% yield, mp 149–153 °C; FT-IR (cm^−1^): 1652, 1596 (C=O), 1349, 1178 (SO_2_). ^1^H NMR (300 MHz, CDCl_3_) *δ* (ppm): 2.96 (m, 4H of piperazine), 3.36 (brs, 4H of piperazine), 4.28 (m, 2H, CH_2_-C=O), 6.96–8.27 (m, 12H, ArH), 15.50 (s, 1H, OH*_enolic_*). ^13^C NMR (300 MHz, CDCl_3_) *δ* (ppm): 187.29, 171.33, 166.97, 164.36, 164.27, 163.59, 138.57, 138.51, 133.33, 132.75, 132.64, 131.84, 131.72, 131.56, 131.52, 129.37, 127.73, 122.34, 121.16, 119.26, 116.49, 116.13, 116.02, 115.84, 51.95, 50.95, 50.71, 44.71, 41.23. HRMS (ESI) calculated for C_27_H_23_F_2_N_3_O_5_S [M + H]^+^ 540.1399; found the following: 540.1371. Analysis calculated for C_27_H_23_F_2_N_3_O_5_S (539.55); C, 60.10; H, 4.30; N, 7.79; found the following: C, 60.10; H, 4.10; N, 7.80.

***Synthesis and properties of series B compounds ***

To the mixture of compound **4** (5 mmol) in 20 mL of anhydrous ethanol with sodium ethoxide (2.3%, 5 mL) was added 5 mmol of 1-(2-chloro-1-oxoethyl)-4-(3,4-dichlorophenyl) piperazine **6** (for compounds **BS130** and **BS230**) or 1-(2-chloro-1-oxoethyl)-4-(*m*-trifluoromethylphenyl)piperazine **7** (for compound **BS62**) and refluxed with stirring for 10–12 h. When the reaction ended, which was controlled on TLC plates, ethanol was distilled off, the residue was treated with 50 mL of chloroform, and insoluble materials were filtered off. The filtrate was then evaporated, and the residue was purified by crystallization from ethanol to give desirable products with 37–67% yields. 

*3-(4-Fluorobenzoyl)-2-{2-[4-(m-trifluoromethylphenyl)-1-piperazinyl]-2-oxoethyl}-4-hydroxy-2H-1,2-benzothiazine 1,1-dioxide* (**BS62**). Orange crystals, 37.50% yield, mp 158–160 °C; FT-IR (cm^−1^): 1652, 1598 (C=O), 1335, 1153 (SO_2_). ^1^H NMR (300 MHz, CDCl_3_) *δ* (ppm): 3.10 (m, 4H of piperazine), 3.40 (brs, 4H of piperazine), 3.70 (m, 2H, CH_2_-C=O), 7.13–8.27 (m, 12H, ArH), 15.50 (brs, 1H, OH*_enolic_*). ^13^C NMR (300 MHz, CDCl_3_) *δ* (ppm): 187.29, 171.34, 164.36, 149.41, 138.49, 133.33, 132.68, 132.05, 131.85, 131.73, 131.54, 130.02, 129.37, 127.75, 125.76, 122.37, 120.27, 118.49, 116.12, 116.02, 115.83, 113.55, 51.91, 49.62, 44.43, 40.95. HRMS (ESI) calculated for C_28_H_23_F_4_N_3_O_5_S [M + H]^+^ 590.1367; found the following: 590.1368. Analysis calculated for C_28_H_23_F_4_N_3_O_5_S (589.56); C, 57.04; H, 3.93; N, 7.13; found the following: C, 57.37; H, 3.80; N, 6.95.

*3-Benzoyl-2-{2-[4-(3,4-dichlorophenyl)-1-piperazinyl]-2-oxoethyl}-4-hydroxy-2H-1,2-benzothiazine 1,1-dioxide* (**BS130**). Yellow crystals, 66.96% yield, mp 146–149 °C; FT-IR (cm^−1^): 1642, 1589 (C=O), 1337, 1176 (SO_2_). ^1^H NMR (300 MHz, CDCl_3_) *δ* (ppm): 2.94–3.24 (m, 8H of piperazine), 3.71–4.08 (m, 2H, CH_2_-C=O), 6.63–8.25 (m, 12H, ArH), 15.58 (brs, 1H, OH*_enolic_*). ^13^C NMR (300 MHz, CDCl_3_) *δ* (ppm): 188.52, 171.70, 164.31, 149.01, 138.39, 135.29, 133.32, 133.12, 132.68, 130.75, 129.43, 129.02, 128.68, 127.75, 122.44, 118.31, 116.23, 52.16, 49.19, 44.41, 40.91. HRMS (ESI) calculated for C_27_H_23_Cl_2_N_3_O_5_S [M + H]^+^ 572.0808; found the following: 572.0807. Analysis calculated for C_27_H_23_Cl_2_N_3_O_5_S (572.46); C, 56.65; H, 4.05; N, 7.34; found the following: C, 56.40; H, 3.90; N, 7.40.

*3-(4-Chlorobenzoyl)-2-{2-[4-(3,4-dichlorophenyl)-1-piperazinyl]-2-oxoethyl}-4-hydroxy-2H-1,2-benzothiazine 1,1-dioxide* (**BS230**). Orange crystals, 63.74% yield, mp 110–113 °C; FT-IR (cm^−1^): 1647, 1584 (C=O), 1339, 1174 (SO_2_). ^1^H NMR (300 MHz, CDCl_3_) *δ* (ppm): 1.90 (brs, 2H, CH_2_-C=O), 3.00–3.25 (m, 8H of piperazine), 6.70–8.27 (m, 11H, ArH), 15.47 (brs, 1H, OH*_enolic_*). ^13^C NMR (300 MHz, CDCl_3_) *δ* (ppm): 187.05, 171.80, 164.30, 149.19, 139.12, 138.49, 133.61, 133.40, 133.13, 132.71, 130.74, 130.58, 129.37, 129.04, 127.79, 124.35, 122.45, 118.29, 116.18, 58.46, 52.08, 48.98, 44.52, 41.05, 23.08, 18.43. HRMS (ESI) calculated for C_27_H_22_Cl_3_N_3_O_5_S [M + H]^+^ 608.0393; found the following: 608.0298. 

### 4.2. Biological Experiments

#### 4.2.1. Cell Lines

An MCF10A non-tumorigenic epithelial cell line (Cat. no. EP-CL-0525) was purchased from Elabscience (Wuhan, China), and MCF7 cells were obtained courtesy of the Department of Basic Medical Sciences, Wroclaw Medical University (Poland).

#### 4.2.2. Cell Culture

MCF10A cells were cultured in DMEM/F12 medium (Dulbecco’s Modified Eagle’s Medium/Nutrient Mixture F-12 Ham, Cat. No. D8062, Sigma-Aldrich, Darmstadt, Germany), 5% horse serum (Cat. No. H1270, Sigma-Aldrich, Darmstadt, Germany), 20 µg/mL recombinant human EGF (Cat. No. PHG0311l, Gibco, ThermoFisher Scientific, Waltham, MA, USA), 0.5 µg/mL hydrocortisone solution (Cat. No. H6909, Sigma-Aldrich, Darmstadt, Germany), 10 µg/mL human insulin solution (Cat. No. I9278, Sigma-Aldrich, Darmstadt, Germany), 1% NEAA (Cat. No. 11140-050, Gibco, Thermo Fisher Scientific, Waltham, MA, USA), 100 µg/mL cholera toxin from *Vibrio cholerae* (Cat. No. C8052, Sigma-Aldrich, Darmstadt, Germany), 1% penicyllin/streptomycin (Cat. No. P4333, Sigma-Aldrich, Darmstadt, Germany). For the MCF7 cells, culture DMEM supllemented with 10% FBS (fetal bovine serum, Cat. No. F7524, Sigma-Aldrich, Darmstadt, Germany) and 1% penicyllin/streptomycin (Cat. No. P4333, Sigma-Aldrich, Darmstadt, Germany) was used. All of the cells were incubated at 37 °C in a 5% CO_2_ atmosphere.

#### 4.2.3. Preparation of Compounds for *In Vitro* Testing

10 mg of each compound was dissolved in 500 µL of DMSO (Cat. No. 113635509, Chempur, Piekary Śląskie, Poland) and then diluted to 10 mg/mL by adding 500 µL of water. To test the effect of the synthesized compounds on cell viability, three different concentrations of compounds were prepared: 10 µM, 20 µM and 50 µM. In addition, the effect of the synthesized compounds in combination with doxorubicin (DOX, Cat. no. D1515, Sigma-Aldrich Darmstadt, Germany) was investigated. For this purpose, mixtures of the previously described compound concentrations were prepared with 1 µM and 5 µM of DOX. All mentioned concentrations were obtained by diluting the compounds and DOX with the appropriate culture medium.

#### 4.2.4. Cell Viability Testing

Cell viability studies were carried out using the MTT assay. A 96-well plate was plated with 4 × 10^3^ cells/well in 200 µL of appropriate culture medium. After 24 h, the cells were treated with different concentrations of compounds and their combinations with DOX. Cells were also treated with 0.6% DMSO to test the effect of the DMSO in which the compounds were dissolved. The control was no-treated cells, cultured in the appropriate culture medium. Treated cells were incubated for 24 h, 48 h, and 72 h. After the appropriate incubation time, 20 µL of 5 mg/mL MTT solution (methylthiazolyldiphenyl-tetrazolium bromide, Cat. No. M2128, Darmstadt, Germany) was added to the wells and incubated for 4 h at 37 °C in a 5% CO_2_ atmosphere. After incubation time, the contents of the wells were removed and 200 µL of DMSO was added. The plate was incubated for 30 min. Absorbance was measured spectrophotometrically at 570 nm and 630 nm.

#### 4.2.5. Statistical Analysis

The experiments were repeated at least three times to ensure accuracy. Statistical analysis was conducted using the *Statistica 13* software from StatSoft (Tulsa, OK, USA) in order to calculate the average values and standard deviation. The Shapiro-Wilk W test was used to check if the data followed a normal distribution. To determine if there were significant differences between groups, the Student’s *t*-test was applied. In all cases, *p* < 0.05 was considered to indicate statistically significant results. If the variables did not meet the assumptions of a normal distribution, the nonparametric Mann–Whitney U test was used.

### 4.3. Fluorescence Studies

The competitive binding of 1,2-benzothiazine derivatives and Hoechst 33342 against ct-DNA has been investigated with fluorescence spectroscopy by keeping a constant concentration of [ct-DNA] = 0.19 µM (in base pair) and [Hoechst] = 15 µM in 10 mM Tris–HCl buffer, pH = 7.2 (ratio of ct-DNA/Hoechst 33342 of 1:79 determined by fluorescence spectroscopy). 1,2-benzothiazine derivatives concentrations in the range 0–100 µM were used. The excitation wavelength was 349 nm and the emission wavelength was observed between 350 nm and 650 nm. Solutions of ct-DNA/Hoechst 33342 without compounds were used as a control. The *Stern–Volmer* equation is as follows [31,32]:

*F*_0_/*F* = 1 + *K*_SV_[Q] = 1 + *k*_q_*τ*_0_[Q] (1)

where *F*_0_ and *F* are the fluorescence intensity in the absence and presence of 1,2-benzothiazine derivatives, respectively; *K*_SV_ is the *Stern–Volmer* quenching constant; *k*_q_ is the quenching rate constant; *τ*_0_ is the average lifetime of the fluorescence molecules without 1,2-benzothiazine derivatives that is 10^−8^ s; and [Q] is 1,2-benzothiazine derivatives concentration. The slope of linear plot of *F*_0_/*F* versus [Q] gives *K*_SV_. For the static quenching interaction, the number of binding sites (*n*) can be determined by the following equation:

log [(*F*_0_ − *F*)/*F*] = log *K* + *n*log [Q] (2)

where *F*_0_ and *F* are the fluorescence intensities in the absence and presence of 1,2-benzothiazine derivatives, respectively, and [Q] is the concentration of 1,2-benzothiazine derivatives.

### 4.4. Molecular Docking Studies

The structure of designed compounds was optimized at the B3LYP/6-31++G** level of theory using the Gaussian16 program [33]. The solvent effects were taken into account by using the PCM method [34]. The molecular docking procedure of ligands to the protein structure was performed in the AutoDock4.2 program [35]. In the present project, we applied the standard protocol of docking. The protein structure of DNA (PDBID:1VZK) and Topo IIα (PDBID:5GWK) was specially prepared [36,37]. The docking procedure was validated by docking tiophene derivatives and etoposides to the crystal structure of DNA and protein, and comparing its binding mode in the original crystallographic structure. The calculated root mean square deviation (RMSD), predicted on the LigRMSD web server, was less than 1 and 1.5 Å, respectively [38]. In the previous studies we described, in detail, the protein preparation and docking procedure [39,40]. The visualization of the obtained results was performed in BIOVIA Discovery Studio (Dassault Systèmes Corporate, Dassault Systèmas, Waltham, MA, USA) and Chimera [41,42].

## 5. Conclusions

This article presents the synthesis and *in vitro* preliminary studies, together with spectroscopic measurements and molecular docking of new phenylpiperazine derivatives of 1,2-benzothiazine with anticancer activity. The tested compounds did not show greater toxicity to healthy cells than the reference drug—doxorubicin in the MTT test. When tested on cancer cells (MCF7), they showed varied cytotoxicity, some of which was greater than doxorubicin, a well-known anticancer drug. The most cytotoxic compound for breast cancer cells, and at the same time with low toxicity to healthy cells, was **BS230**, containing a 1-(3,4-dichlorophenyl)piperazine substituent in its structure. All compounds also showed the ability to bind to the minor groove of ct-DNA in the spectroscopic study. Molecular docking to the binding pocket of topoisomerase IIα confirmed that the tested compounds interact with this enzyme, with **BS230** being the most potent, confirming the *in vitro* studies. Summarizing, the results suggest that proposed new 1,2-benzothiazine derivatives are promising, safe candidates that could be considered for further research into effective anticancer therapy.

## Figures and Tables

**Table 1:** Binding parameters obtained from the fluorescence quenching method; pH = 7.2; T = 25 °C.

Compound	K_sv_ (1 × 10^3^) (M^−1^)	k_q_ (1 × 10^11^) (M^−1^ s^−1^)	K (1 × 10^3^) (M^−1^)	*n*	R^2^
**BS62**	2.16	2.16	2.34	0.92	0.995
**BS130**	3.32	3.32	3.21	0.99	0.997
**BS133**	1.35	1.35	0.61	1.02	0.994
**BS230**	2.41	2.41	2.86	1.01	0.996
**BS233**	1.98	1.98	2.13	0.97	0.997
**BS433**	3.23	3.23	2.64	0.99	0.995
**BS533**	3.06	3.06	3.96	0.99	0.995
**BS633**	3.41	3.41	3.12	1.01	0.998

**Table 2:** Free energy of binding of designed compounds to DNA and topoisomerase IIα.

Compound	∆G_binding_ to DNA [kJ/mol]	∆G_binding_ to Topo IIα [kJ/mol]
**BS62**	−61.1	−63.2
**BS130**	−61.3	−63.2
**BS133**	−57.1	−63.1
**BS230**	−63.2	−66.6
**BS233**	−60.2	−64.2
**BS433**	−62.2	−65.6
**BS533**	−58.7	−64.1
**BS633**	−58.4	−63.0
etoposide	−48.3	−44.6

## Data Availability

Data are contained within the article and Supplementary Materials.

## Supplementary Materials

The following supporting information can be downloaded at: https://www.mdpi.com/article/10.3390/molecules29184282/s1, Figures S1–S16—^1^H NMR and ^13^C NMR spectra of the new compounds; Figures S17–S23—Computational studies; Figures S24–S30—*In vitro* anti-proliferative activity.
