# Supplementary material for: Rational Design, Synthesis, Molecular Docking, and Biological Evaluations of New Phenylpiperazine Derivatives of 1,2-Benzothiazine as Potential Anticancer Agents
Source: Molecules. 2024 Sep 10;29(18):4282. doi: 10.3390/molecules29184282 (PMC11433925; doi:10.3390/molecules29184282)
Supplement: Supplementary file 1 [file molecules-29-04282-s001.zip › molecules-3168277-SI.pdf]

Supplementary materials

Rational design, synthesis, molecular docking and biological evaluations of new phenylpiperazine derivatives of 1,2-benzothiazine as potential anticancer agents

Berenika M. Szczęśniak-Sięga[1,*], Natalia Zaręba[2], Żaneta Czyżnikowska[3], Tomasz Janek[4], Marta Kepinska[2]

[1] Department of Medicinal Chemistry, Faculty of Pharmacy, Wroclaw Medical University, Borowska 211, 50-556 Wroclaw, Poland; berenika.szczesniak-siega@umw.edu.pl

[2] Department of Pharmaceutical Biochemistry, Faculty of Pharmacy, Wroclaw Medical University, Borowska 211a, 50-556 Wroclaw, Poland; natalia.zareba@umw.edu.pl, marta.kepinska@umw.edu.pl

[3] Department of Basic Chemical Sciences, Wroclaw Medical University, Borowska 211a, 50-556 Wrocław, Poland; zaneta.czyznikowska@umw.edu.pl

[4] Department of Biotechnology and Food Microbiology, Faculty of Biotechnology and Food Science, Wrocław University of Environmental and Life Sciences, Chełmońskiego 37, 51-630 Wrocław, Poland; tomasz.janek@upwr.edu.pl

Content:

1. **^{1}H NMR** and **^{13}C NMR** spectra of new compounds (pages S3-S10).

2. Computational studies (pages S11-S13).

3. *In vitro* anti-proliferative activity (pages S14-S15).

^{1}H NMR and ^{13}C NMR spectra of new compounds

compound	structure	page
**BS 62**	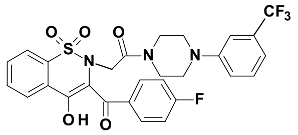	S3
**BS 130**	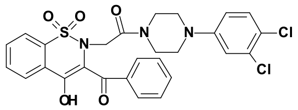	S4
**BS 230**	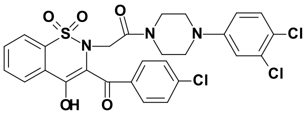	S5
**BS 133**	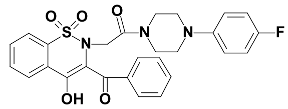	S6
**BS 233**	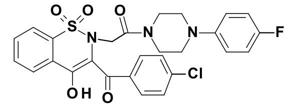	S7
**BS 433**	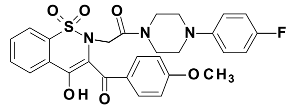	S8
**BS 533**	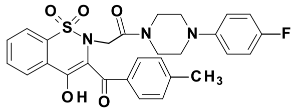	S9
**BS 633**	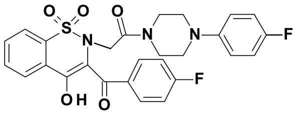	S10

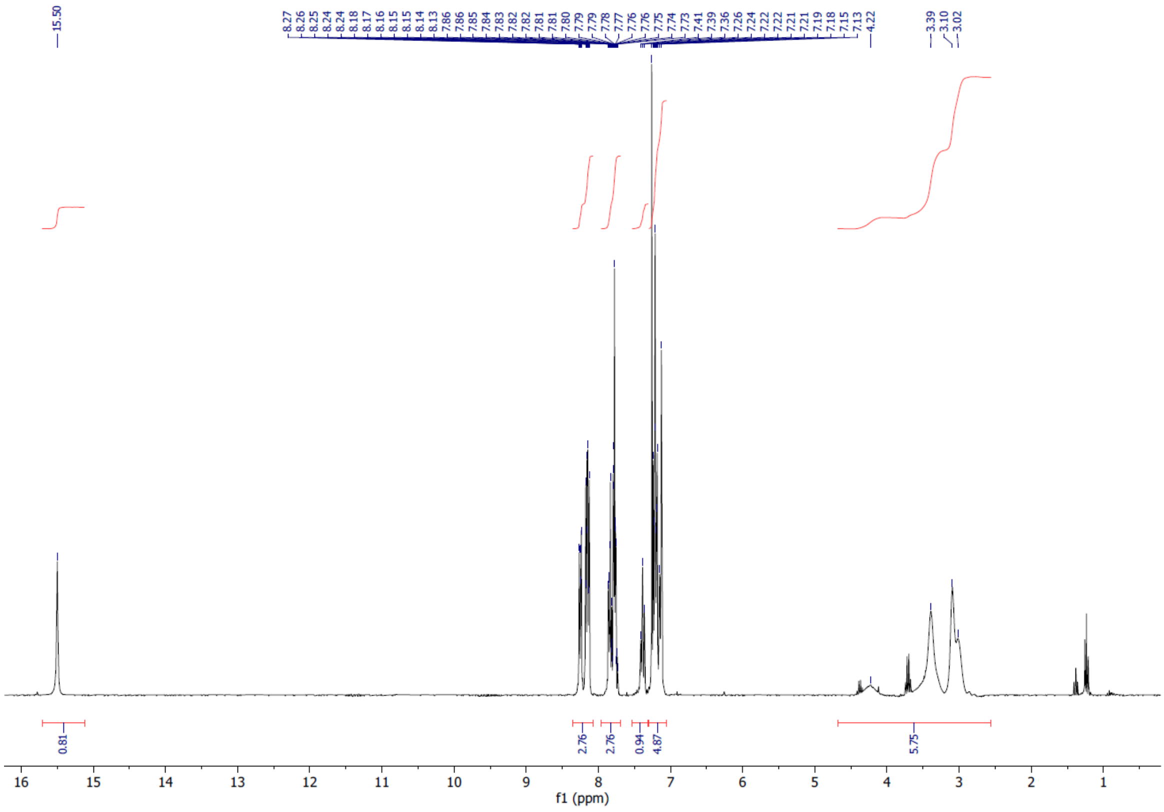

Figure S1. ^{1}H NMR spectrum of **BS 62** in $CDCl_3$.

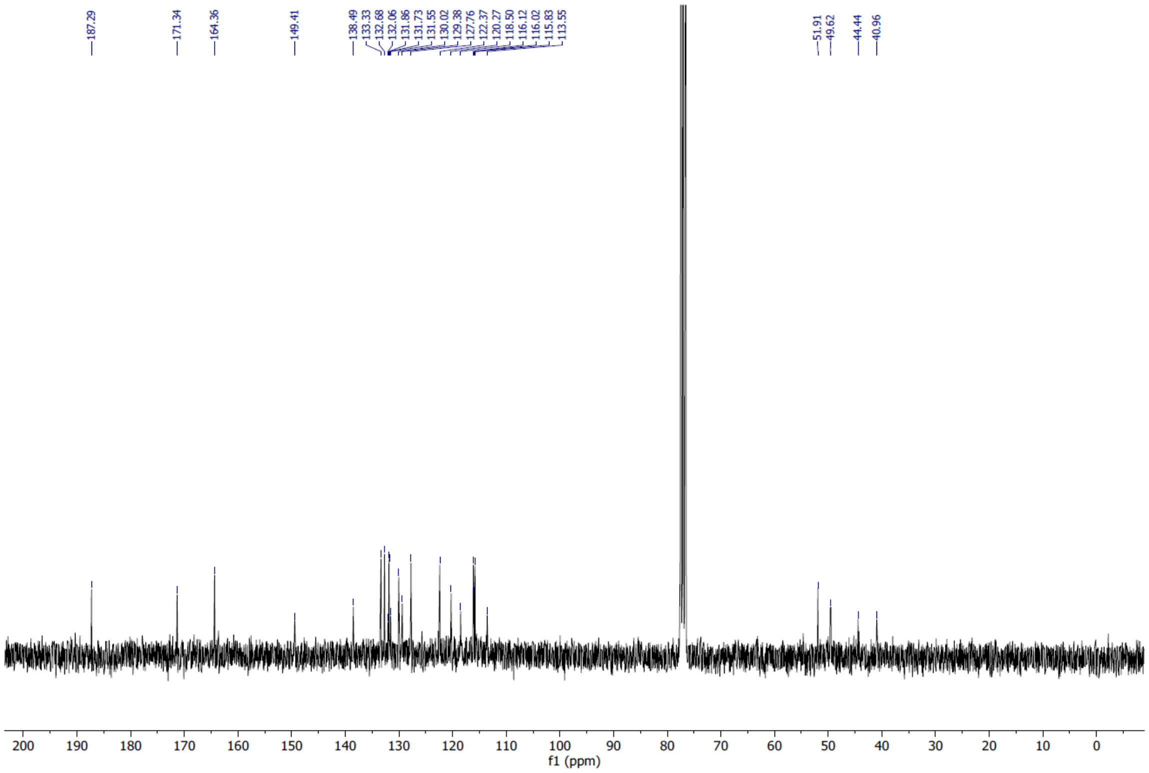

Figure S2. ^{13}C NMR spectrum of **BS 62** in $CDCl_3$.

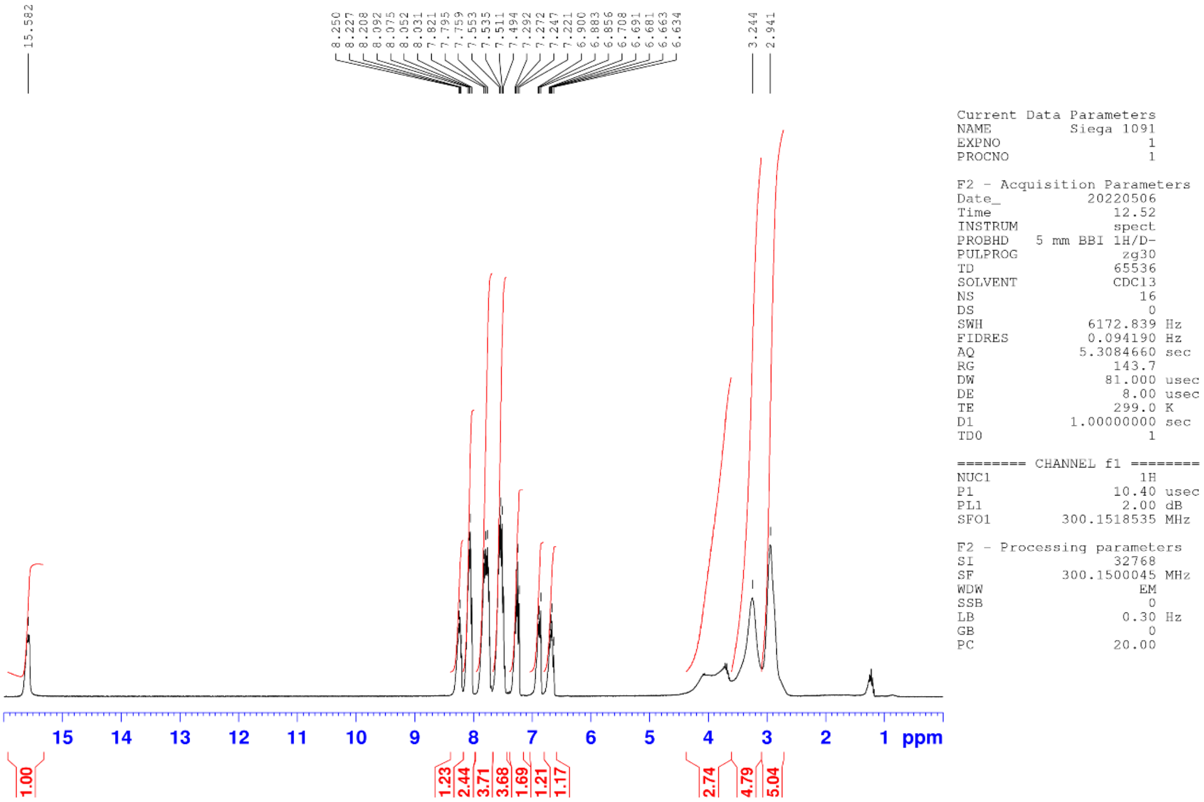

Figure S3. ^{1}H NMR spectrum of **BS 130** in $CDCl_3$.

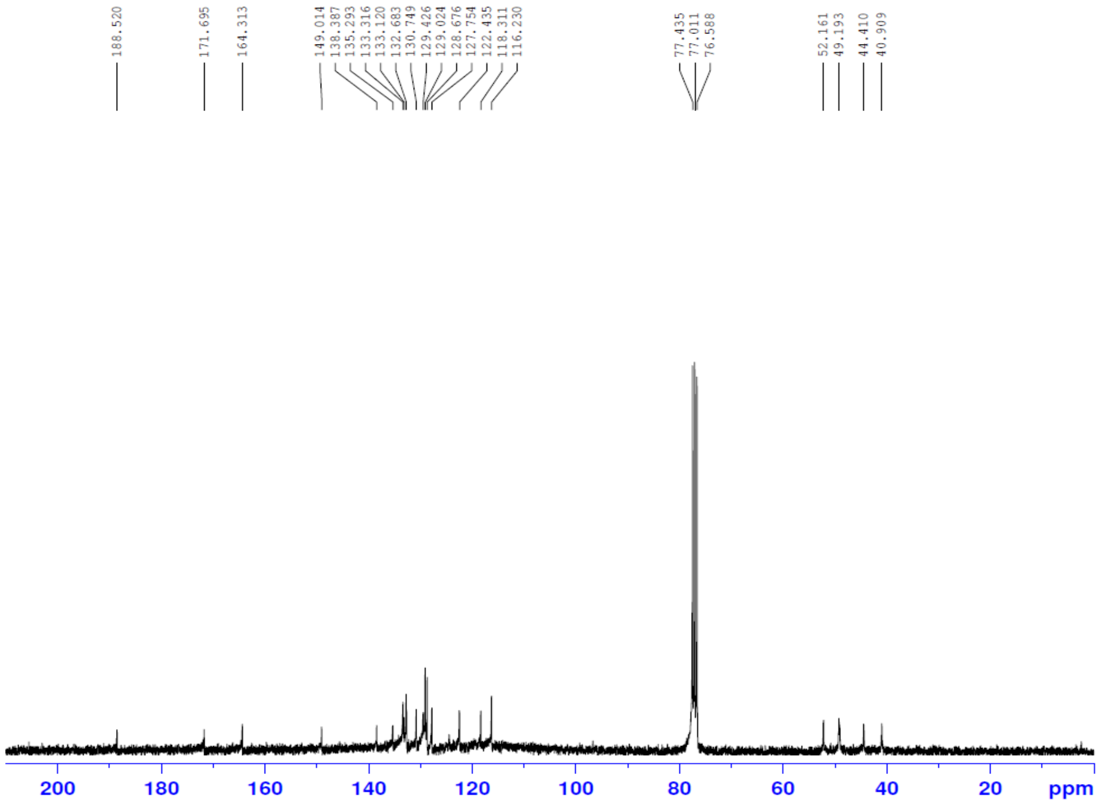

Figure S4. ^{13}C NMR spectrum of **BS 130** in $CDCl_3$.

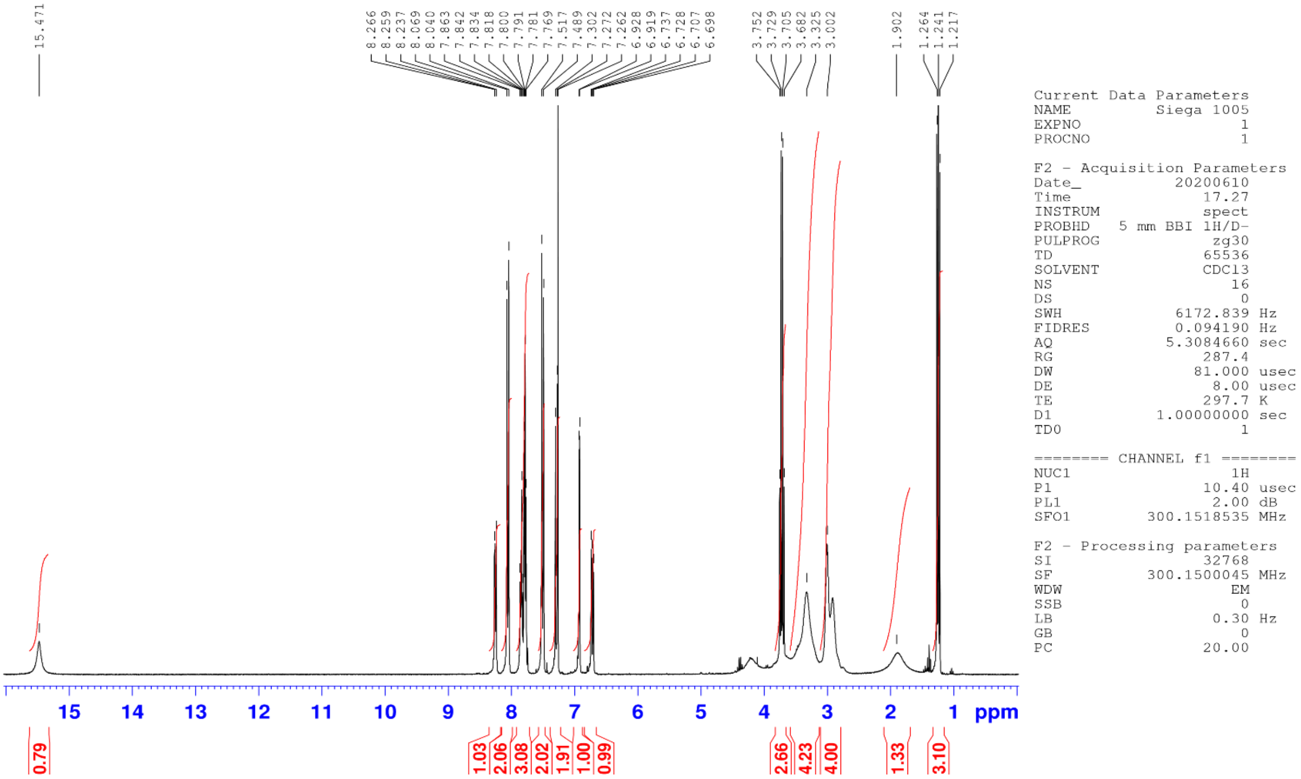

Figure S5. ^{1}H NMR spectrum of **BS 230** in $CDCl_3$.

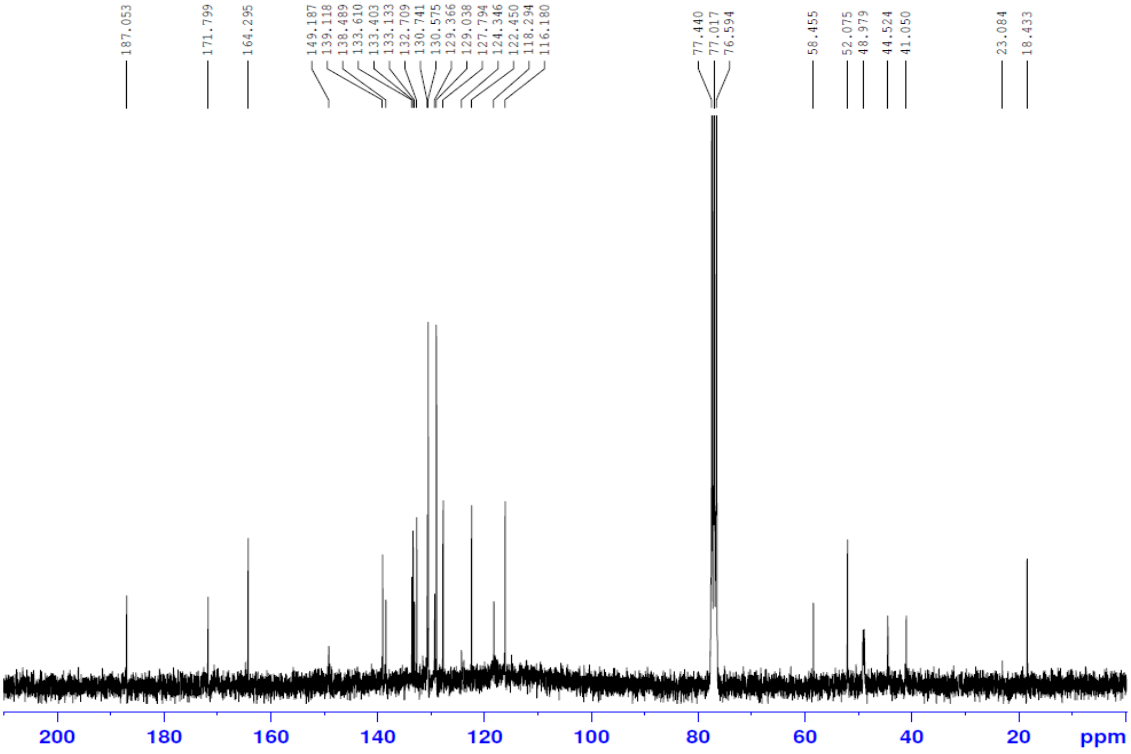

Figure S6. ^{13}C NMR spectrum of **BS 230** in $CDCl_3$.

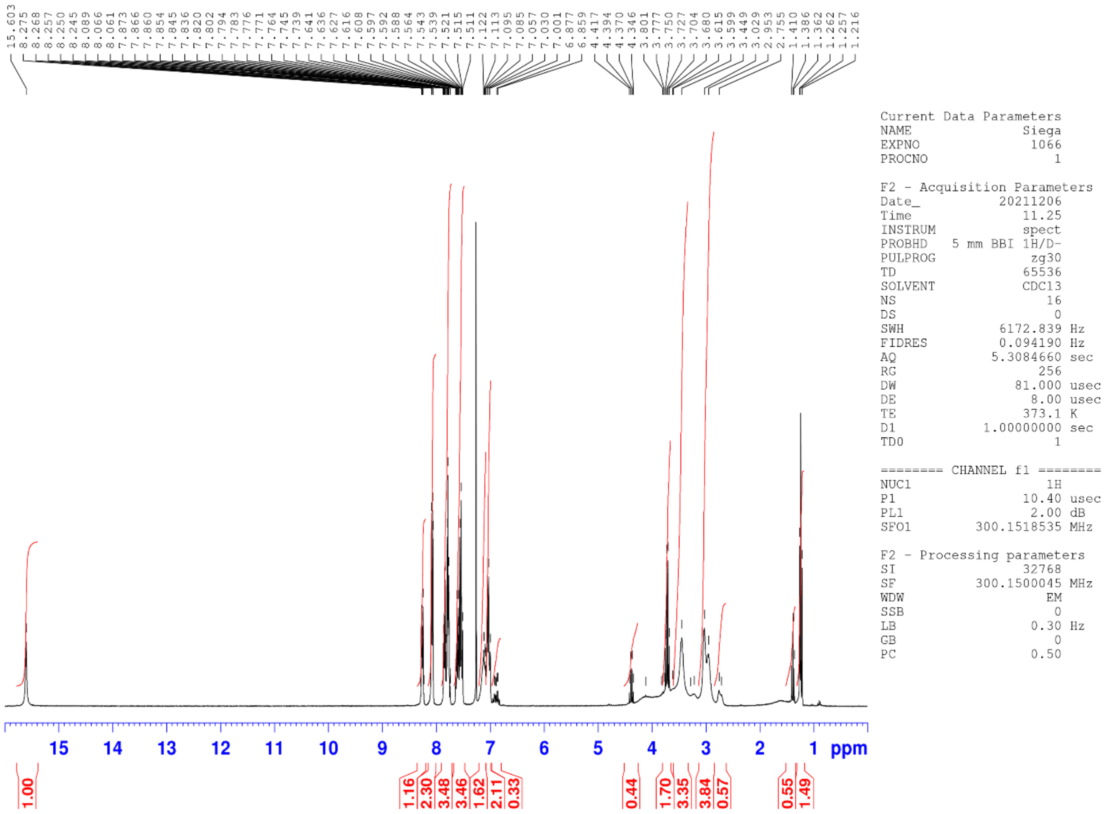

Figure S7. ^{1}H NMR spectrum of **BS 133** in $CDCl_3$.

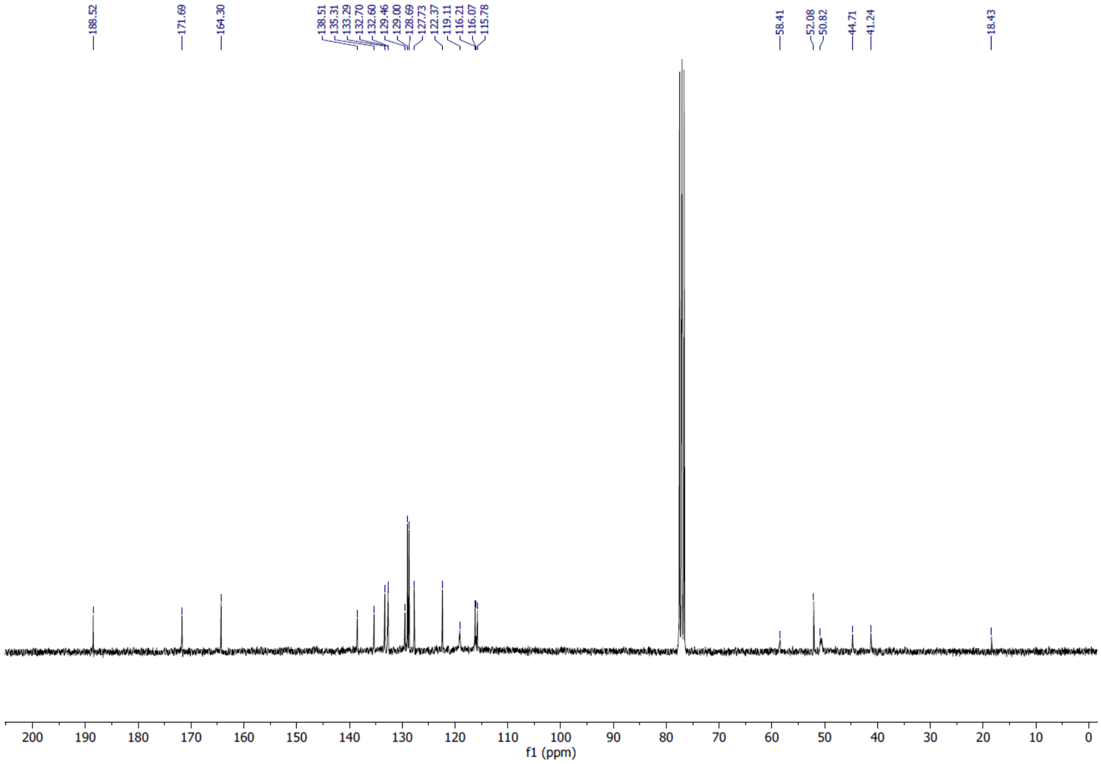

Figure S8. ^{13}C NMR spectrum of **BS 133** in $CDCl_3$.

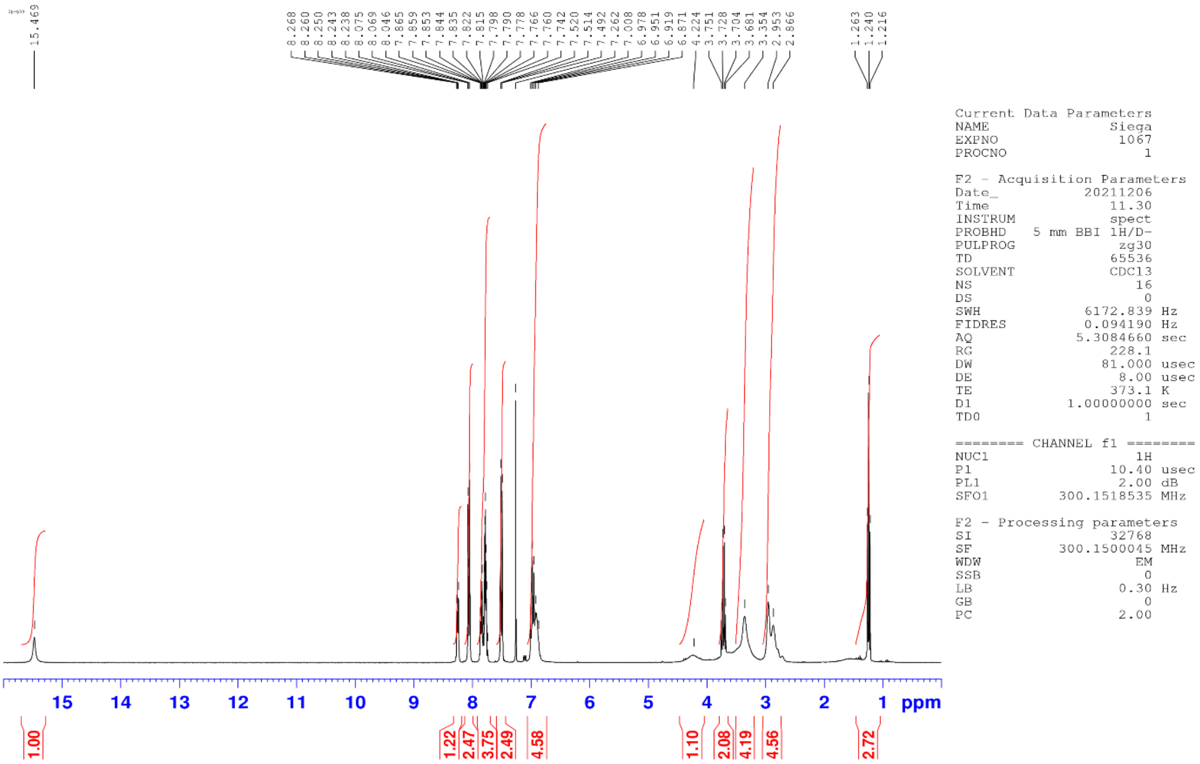

Figure S9. ^{1}H NMR spectrum of **BS 233** in $CDCl_3$.

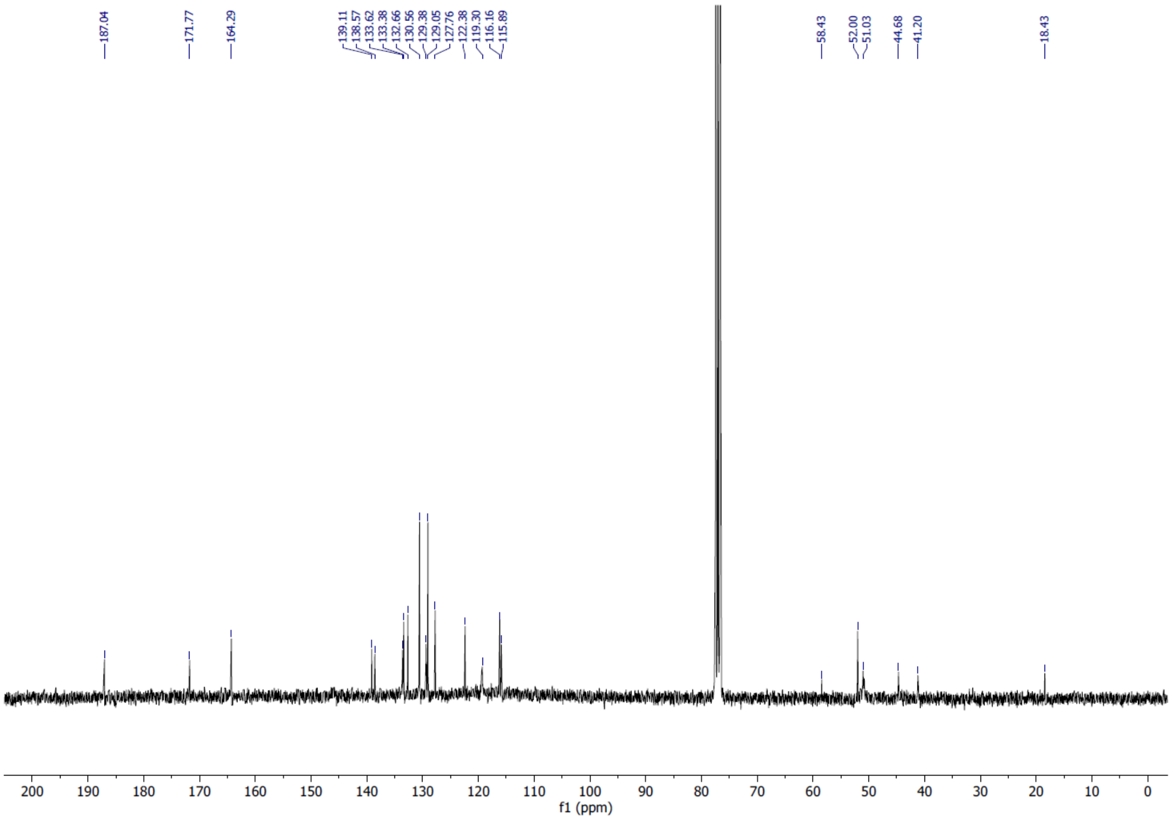

Figure S10. ^{13}C NMR spectrum of **BS 233** in $CDCl_3$.

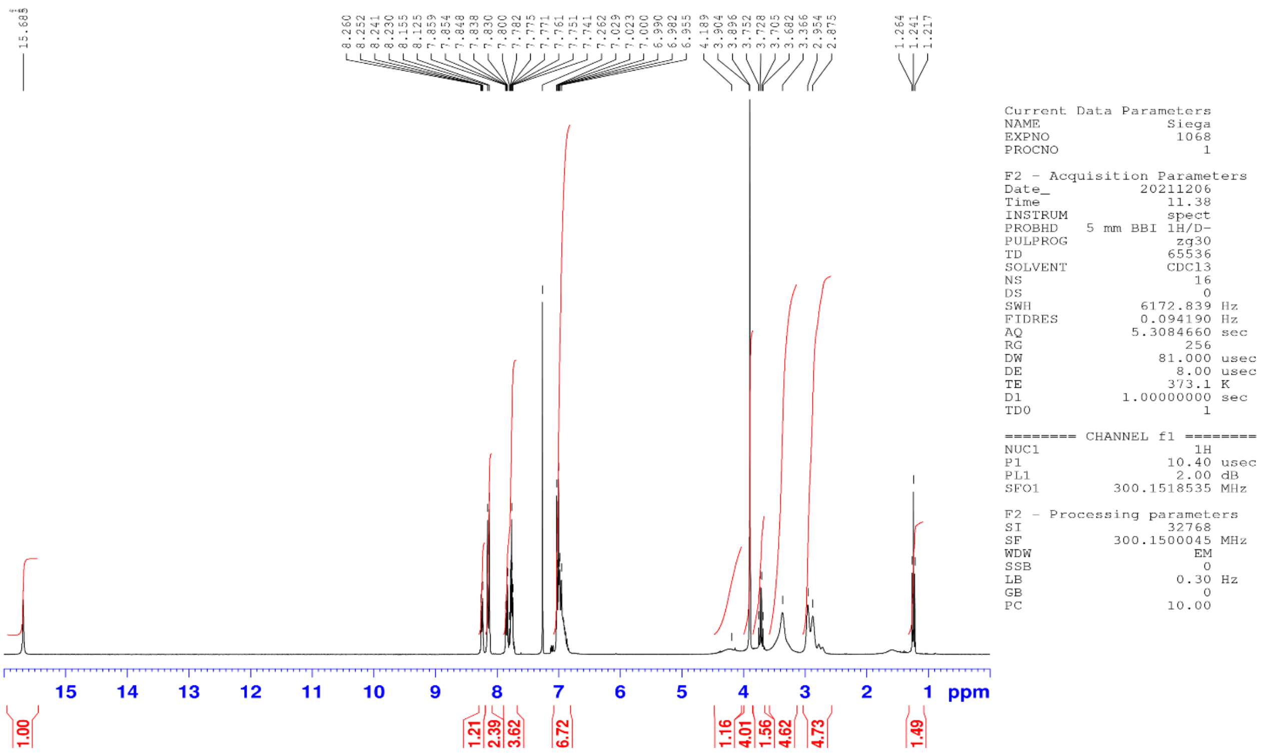

Figure S11. ^{1}H NMR spectrum of **BS 433** in $CDCl_3$.

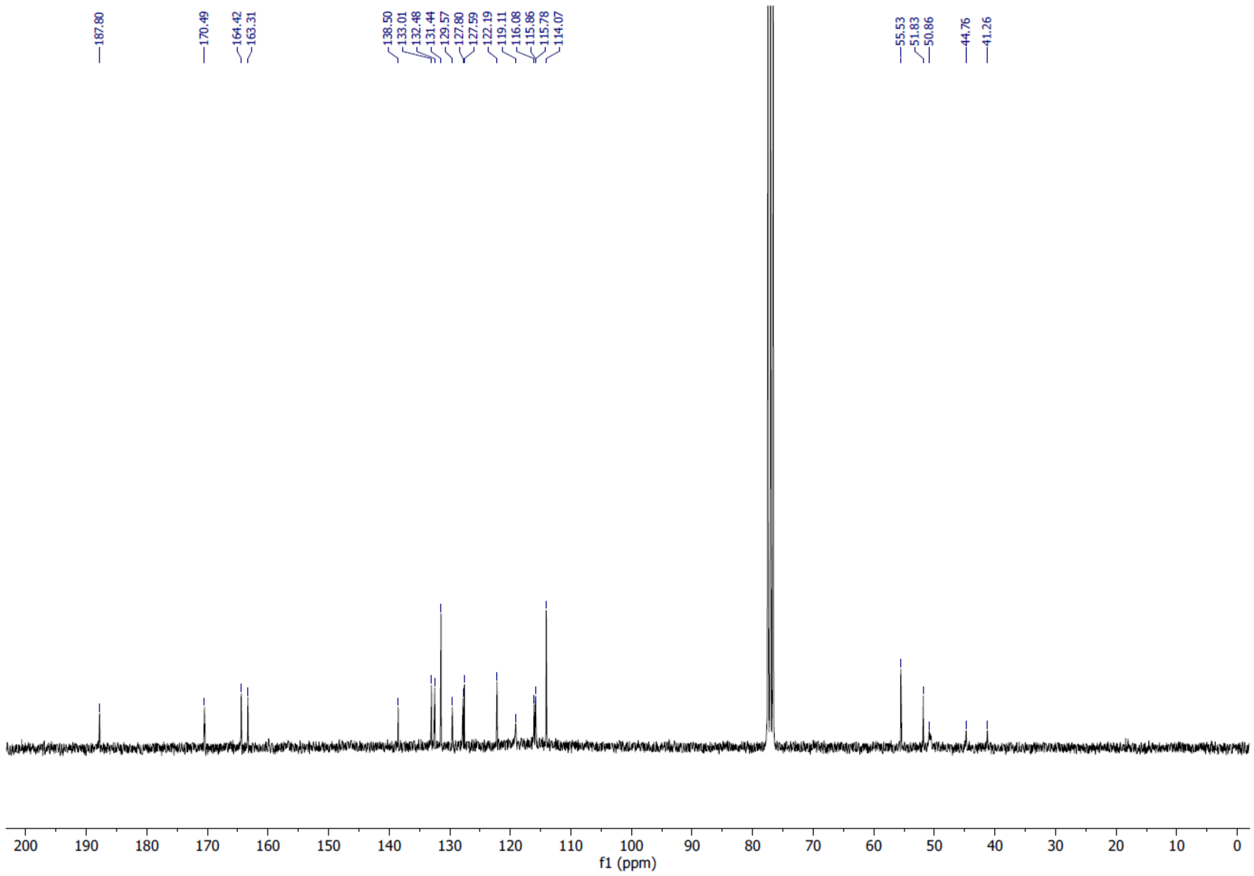

Figure S12. ^{13}C NMR spectrum of **BS 433** in $CDCl_3$.

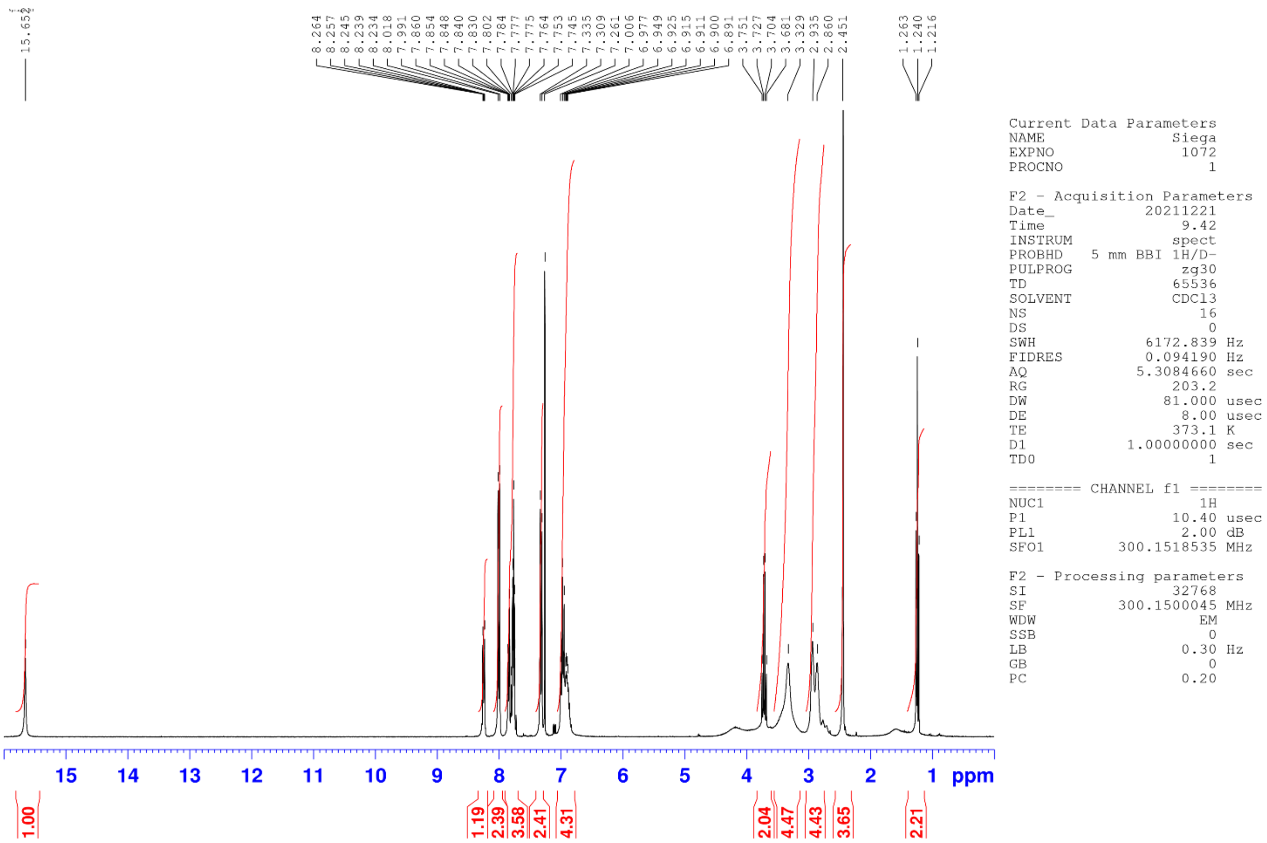

Figure S13. ^{1}H NMR spectrum of **BS 533** in $CDCl_3$.

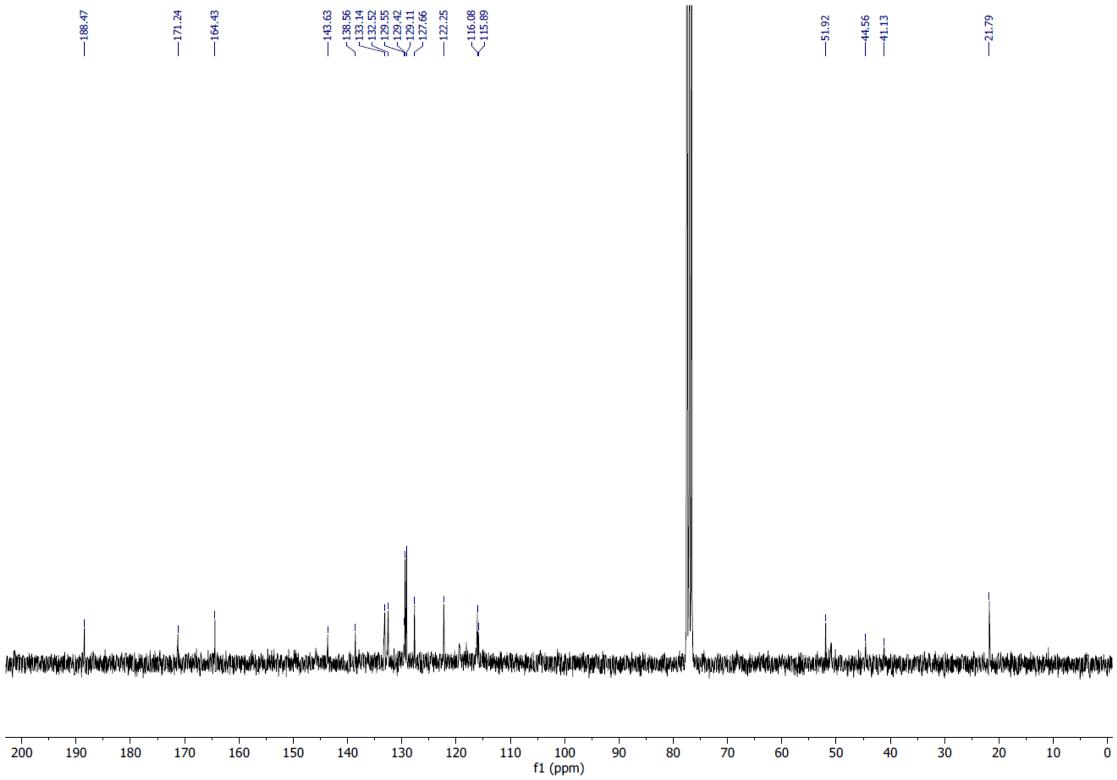

Figure S14. ^{13}C NMR spectrum of **BS 533** in $CDCl_3$.

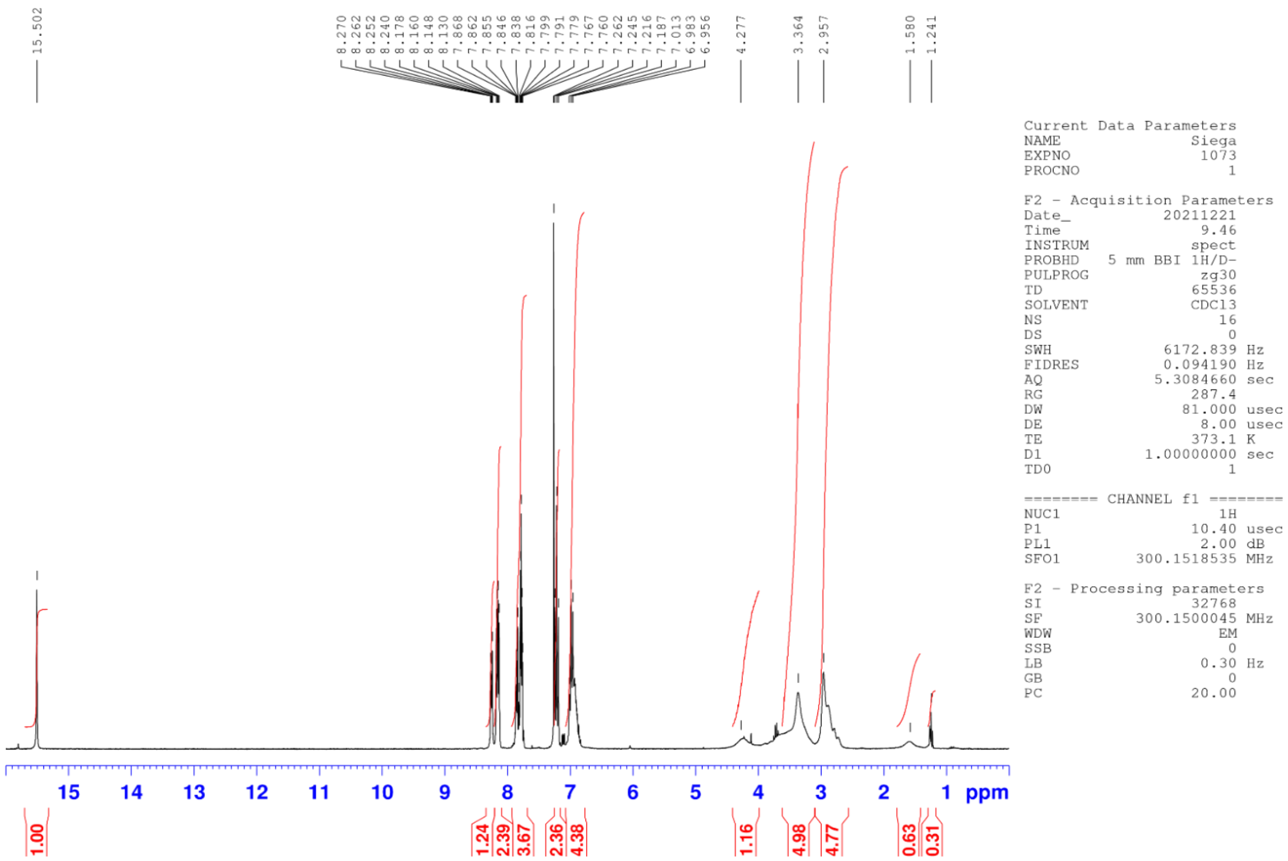

Figure S15. ^{1}H NMR spectrum of **BS 633** in $CDCl_3$.

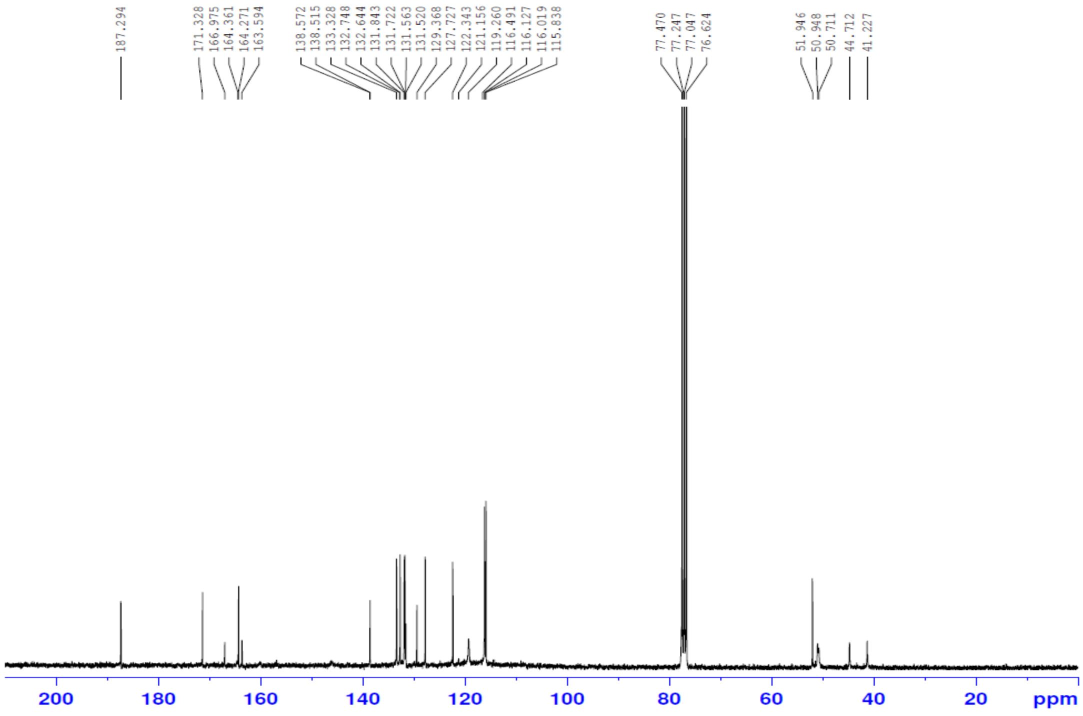

Figure S16. ^{13}C NMR spectrum of **BS 633** in $CDCl_3$.

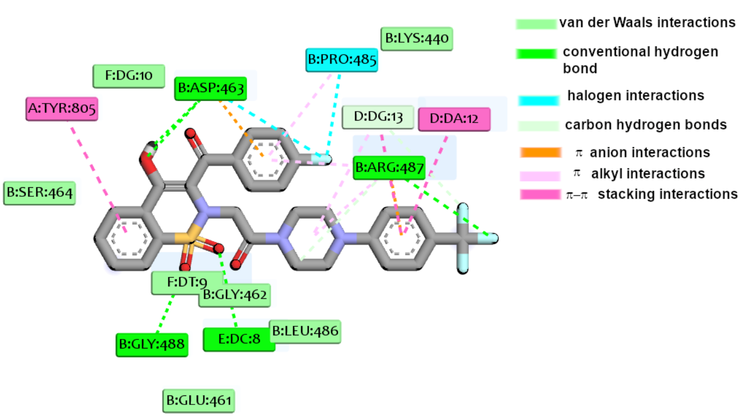

Figure S17. Intermolecular interactions of **BS 62** in the active site of Topo IIα (2D representation).

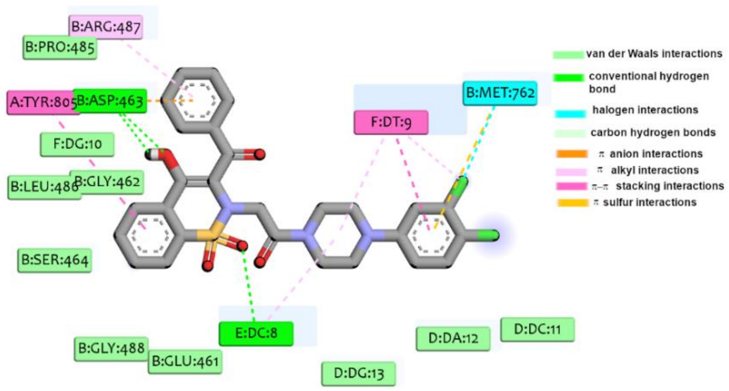

Figure S18. Intermolecular interactions of **BS 130** in the active site of Topo IIα (2D representation).

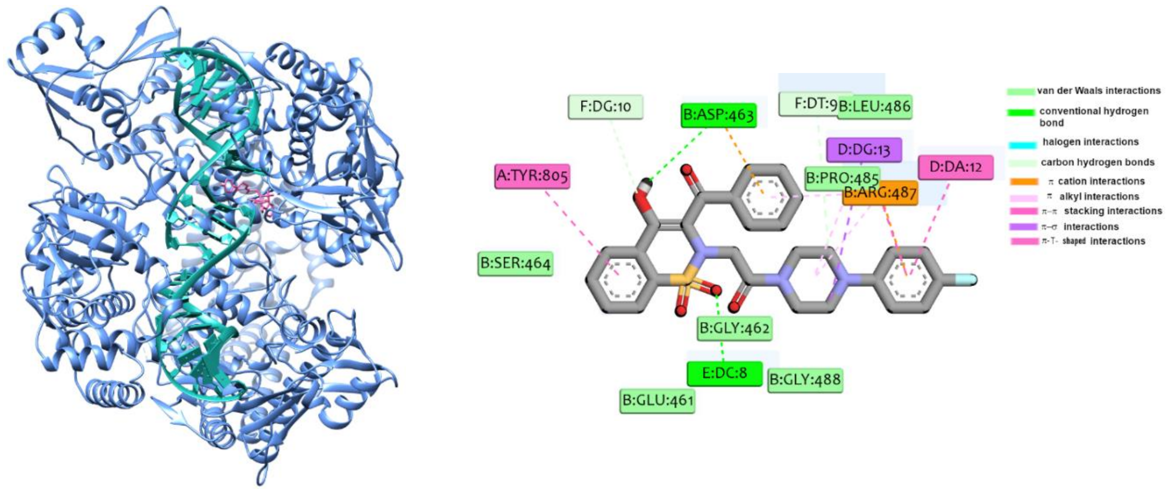

a **b**

Figure S19. a) The binding mode of **BS 133** in the active site of protein. b) Intermolecular interactions of **BS 133** in the active site of Topo IIα (2D representation).

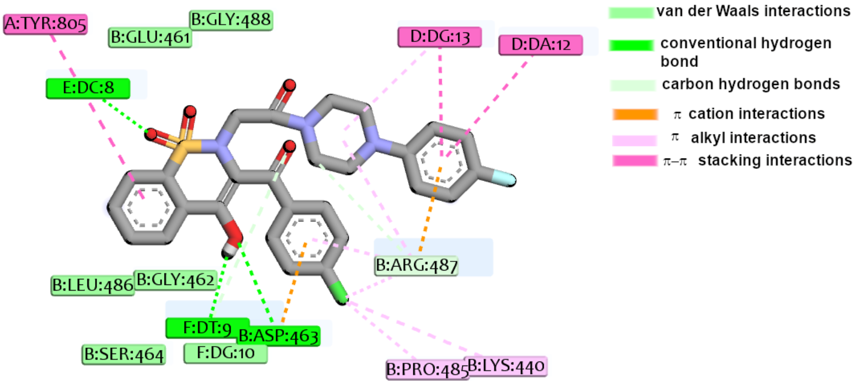

Figure S20. Intermolecular interactions of **BS 233** in the active site of Topo IIα (2D representation).

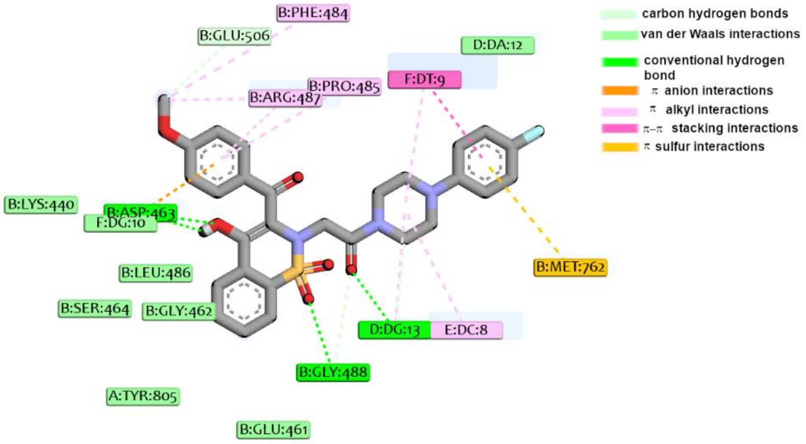

Figure S21. Intermolecular interactions of **BS 433** in the active site of Topo IIα (2D representation).

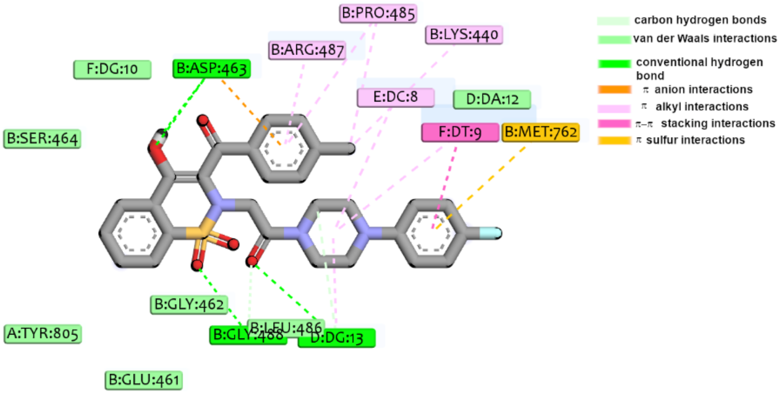

Figure S22. Intermolecular interactions of **BS 533** in the active site of Topo IIα (2D representation).

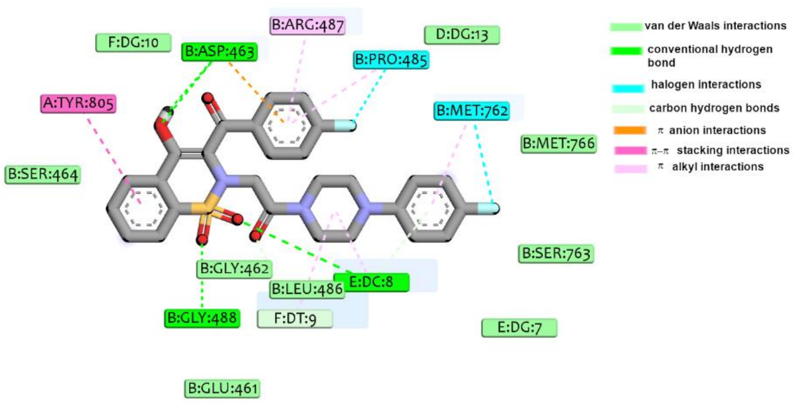

Figure S23. Intermolecular interactions of **BS 633** in the active site of Topo IIα (2D representation).

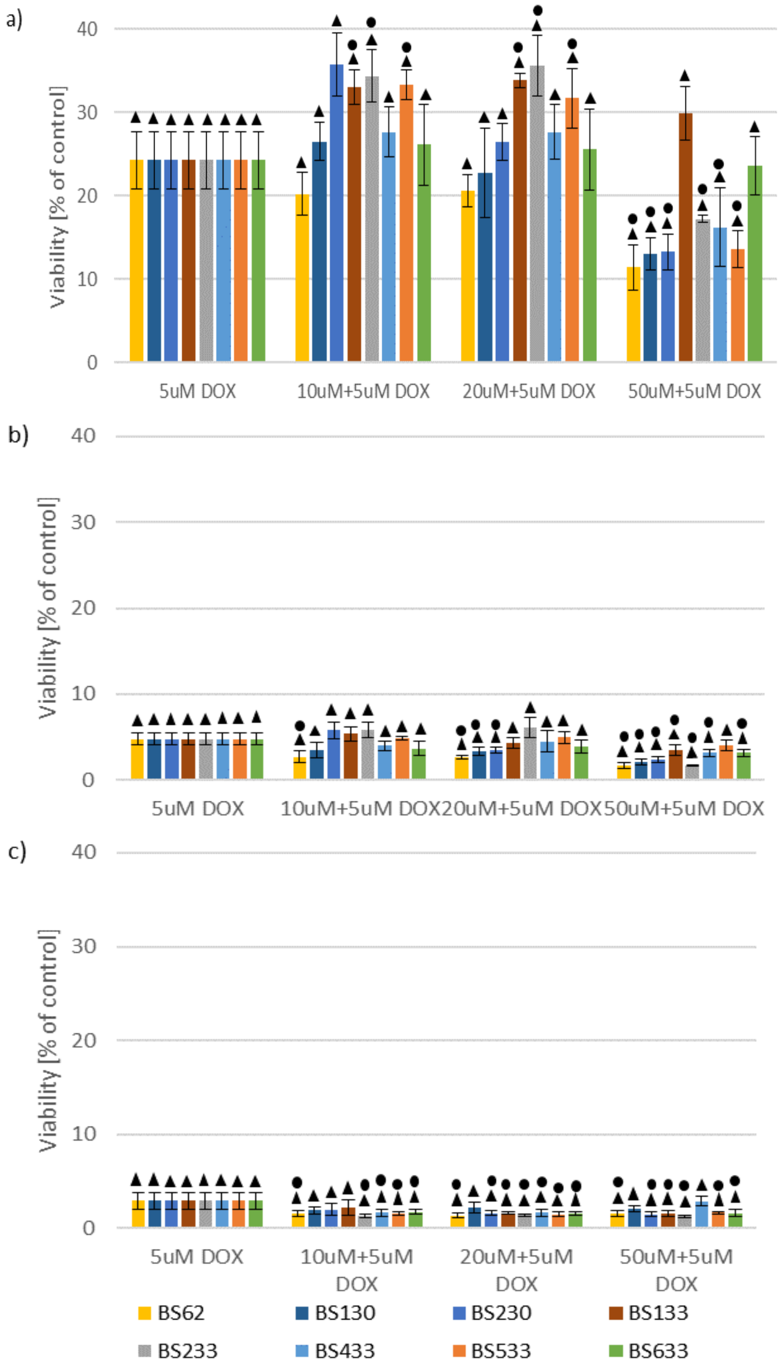

Figure S24. Viability of MCF10A cells treated with **BS** compounds in combination with DOX (5 µM) for (a) 24h, (b) 48h and (c) 72h. The triangle indicates statistical significance compared to the non-treated cells. The dot indicates statistical significance compared to the respective cells treated with 5 µM DOX.

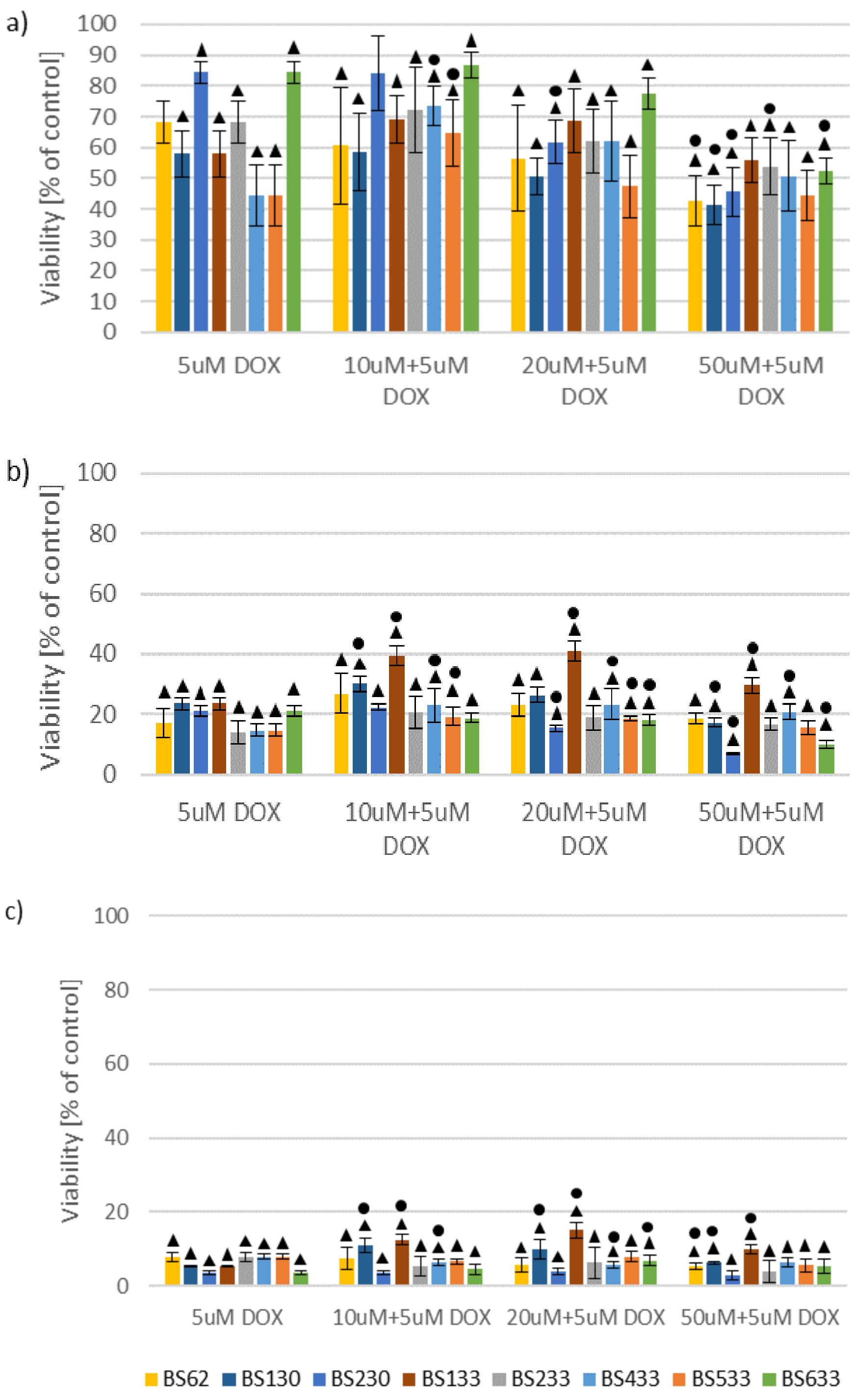

Figure S25. Viability of MCF7 cells treated with **BS** compounds in combination with DOX (5 µM) for (a) 24h, (b) 48h and (c) 72h. The triangle indicates statistical significance compared to the non-treated cells. The dot indicates statistical significance compared to the respective cells treated with 5 µM DOX.

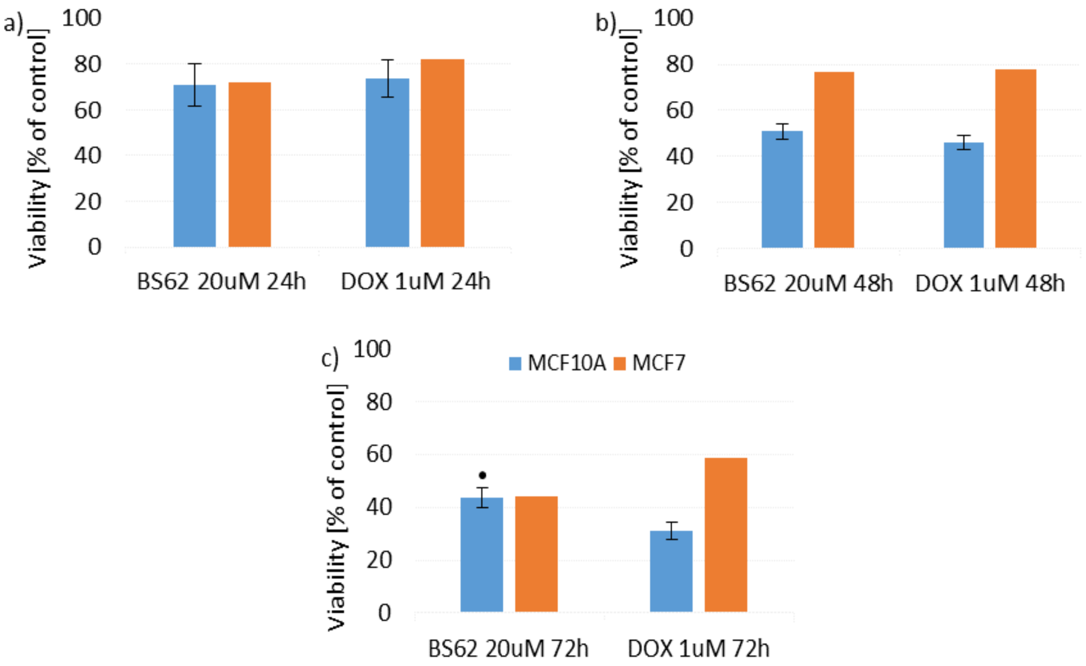

Figure S26. Viability of MCF10A and MCF7 cells treated with **BS62** 20µM compound compared to corresponding cells treated with 1µM DOX for (a) 24h, (b) 48h and (c) 72h. The dot indicates statistical significance compared to the respective cells treated with 1µM DOX.

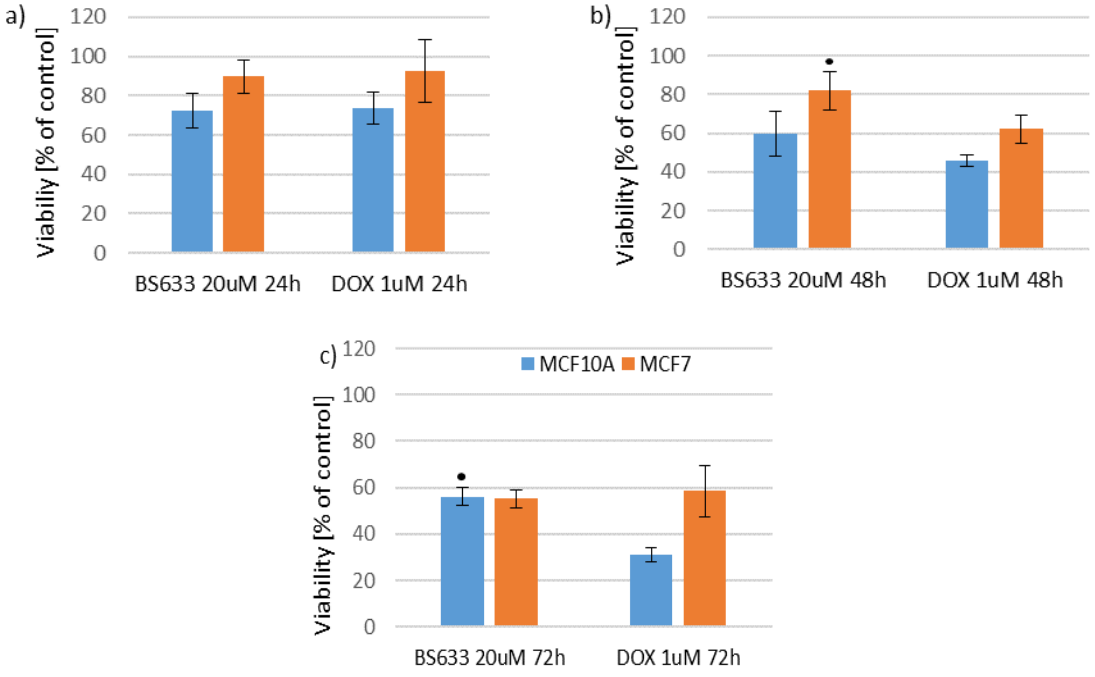

Figure S27. Viability of MCF10A and MCF7 cells treated with **BS633** 20µM compound compared to corresponding cells treated with 1µM DOX for (a) 24h, (b) 48h and (c) 72h. The dot indicates statistical significance compared to the respective cells treated with 1µM DOX.

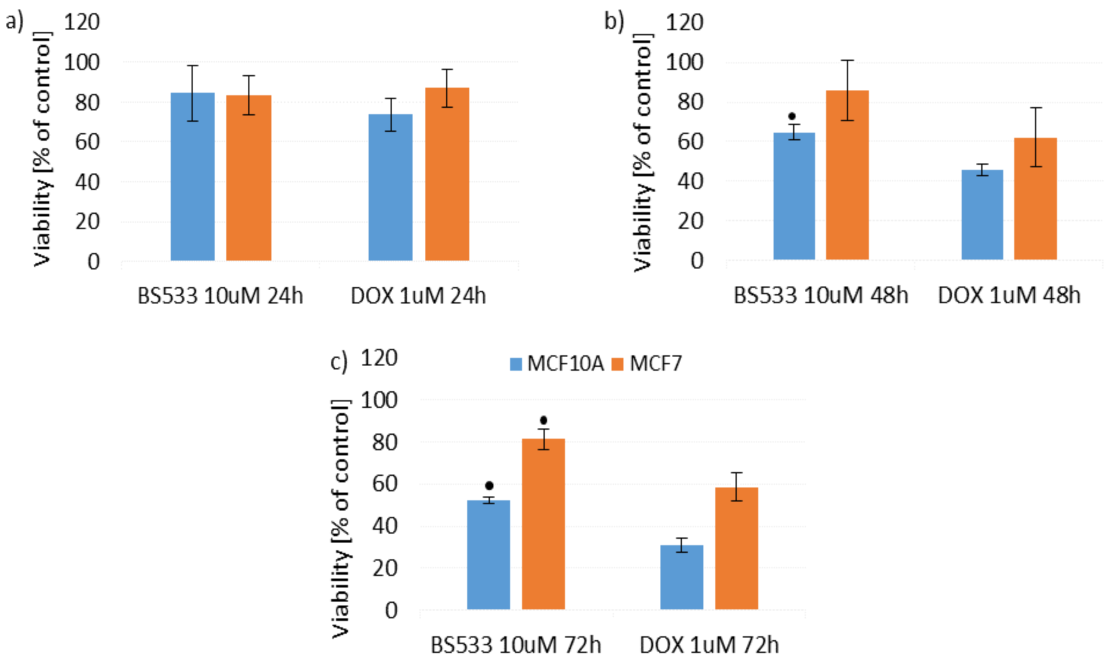

Figure S28. Viability of MCF10A and MCF7 cells treated with **BS533** 10µM compound compared to corresponding cells treated with 1µM DOX for (a) 24h, (b) 48h and (c) 72h. The dot indicates statistical significance compared to the respective cells treated with 1µM DOX.

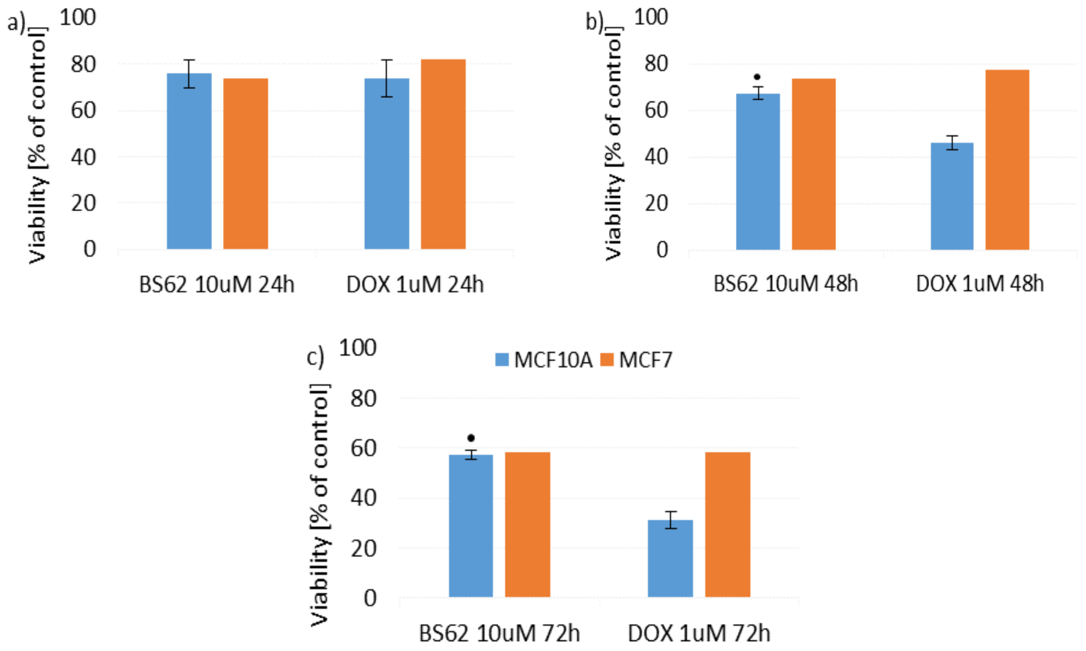

Figure S29. Viability of MCF10A and MCF7 cells treated with **BS62** 10µM compound compared to corresponding cells treated with 1µM DOX for (a) 24h, (b) 48h and (c) 72h. The dot indicates statistical significance compared to the respective cells treated with 1µM DOX.

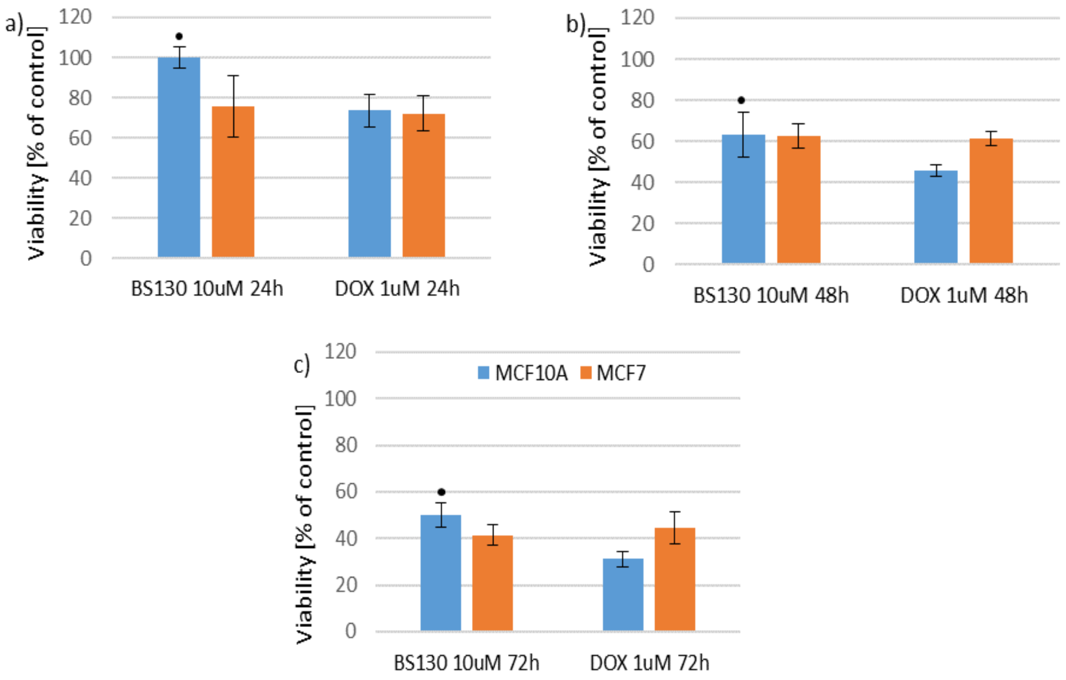

Figure S30. Viability of MCF10A and MCF7 cells treated with **BS130** 10µM compound compared to corresponding cells treated with 1µM DOX for (a) 24h, (b) 48h and (c) 72h. The dot indicates statistical significance compared to the respective cells treated with 1µM DOX.
